# *Akkermansia muciniphila*‐Derived *N*‐Acetylspermidine Modulates the Localization of Intestinal α1,2‐Fucosylated Proteins to Maintain Gut Homeostasis

**DOI:** 10.1002/advs.202506576

**Published:** 2025-08-07

**Authors:** Ye Yao, Zhangming Pei, Yuanyuan Dai, Yinghan Chen, Zepeng Chang, Hongchao Wang, Jianxin Zhao, Hao Zhang, Qixiao Zhai, Wenwei Lu, Wei Chen

**Affiliations:** ^1^ State Key Laboratory of Food Science and Resources Jiangnan University Wuxi 214122 China; ^2^ School of Food Science and Technology Jiangnan University Wuxi 214122 China; ^3^ Department of Gastroenterology Affiliated Hospital of Jiangnan University Wuxi 214122 China; ^4^ School of Biotechnology Jiangnan University Wuxi 214122 China; ^5^ MOE Medical Basic Research Innovation Center for Gut Microbiota and Chronic Diseases School of Medicine Jiangnan University Wuxi 214122 China; ^6^ National Engineering Research Center for Functional Food Jiangnan University Wuxi 214122 China

**Keywords:** α1,2‐fucosylation, *Akkermansia muciniphila*, C1GALT1C1, colitis, HDAC2, protein localization

## Abstract

The growing incidence of inflammatory bowel diseases, including colitis and Crohn's disease, poses a critical challenge for global healthcare. Current development of α1,2‐fucosylation‐enhancing strategies shows significant potential as a colitis treatment modality by promoting gut homeostasis. Although certain probiotics alleviate colitis by enhancing intestinal α1,2‐fucosylation, the molecular mechanisms by which probiotics‐derived metabolites modulate this process remain unclear. This study found that the probiotic *Akkermansia muciniphila* (*A. muciniphila*) enhanced intestinal α1,2‐fucosylation, a crucial factor contributing to its colitis‐alleviating effects. Specifically, *A. muciniphila*‐derived N‐acetylspermidine upregulated α1,2‐fucosylation, thereby enhancing barrier integrity and suppressing inflammation, which are reversed upon α1,2‐fucosylation inhibition. Mechanistically, N‐acetylspermidine upregulated *HDAC2* via *PIM1* inhibition, leading to decreased chromatin accessibility at the *TP73* locus, subsequently increasing the expression of α1,2‐fucosylation‐associated gene *C1GALT1C1*. Furthermore, N‐acetylspermidine‐induced α1,2‐fucosylation enhancement facilitated the membrane localization of ZO‐1 and ZO‐2, while suppressing C3 secretion, both of which contributed to colitis alleviation. Together, our findings elucidate how *A. muciniphila* and its metabolite N‐acetylspermidine regulate intestinal α1,2‐fucosylation to maintain gut homeostasis and highlight their therapeutic potential in developing biological therapies for colitis.

## Graphical abstract

This study demonstrates that *Akkermansia muciniphila* alleviates colitis by enhancing intestinal α1,2‐fucosylation through its metabolite N‐acetylspermidine. Mechanistically, N‐acetylspermidine‐induced *PIM1* inhibition promotes *HDAC2*‐mediated reduction of chromatin accessibility at *TP73*, thereby upregulating *C1GALT1C1* to boost α1,2‐fucosylation. These findings underscore the therapeutic promise of probiotic‐derived α1,2‐fucosylation modulation for inflammatory bowel disease. The graphical abstract is created using BioRender.

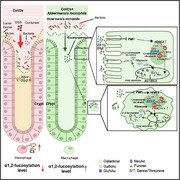

## 1 Introduction

Inflammatory bowel disease (IBD), encompassing Crohn's disease (CD) and ulcerative colitis (UC), is a chronic relapsing inflammatory disorder characterized by mucosal inflammation and dysregulated glycosylation patterns.^[^ ^1^ ^]^ It is becoming a global health challenge due to its escalating incidence.^[^ ^2^ ^]^ The pathogenesis of IBD involves a complex interplay of genetic predisposition, gut microbiota dysbiosis, and aberrant immune responses, all influenced by various glycoproteins.^[^ ^1^, ^3^, ^4^ ^]^ Intestinal epithelial glycosylation is essential for maintaining a balanced interaction between the gut epithelium and microbiota.^[^ ^1^, ^5^ ^]^ Recent studies highlight the distinct roles of epithelial glycosylation in colitis onset and progression. For example, FUT8‐catalyzed α1,6‐fucosylation exacerbates UC by altering the quantity and quality of secreted mucins.^[^ ^6^ ^]^ In contrast, FUT2‐mediated α1,2‐fucosylation enhances the intestinal barrier, and alleviates colitis.^[^ ^7^ ^]^ However, the underlying mechanisms by which epithelial α1,2‐fucosylated proteins regulate these processes remain poorly investigated.

Germ‐free mice exhibit defective intestinal α1,2‐fucosylation, which can be restored by colonization with conventional microbiota or the bacterium *Bacteroides thetaiotaomicron*.^[^ ^8^ ^]^ These findings suggest that gut microbiota or their derived signals play a crucial role in inducing host α1,2‐fucosylation to alleviate intestinal damage.^[^ ^8^, ^9^ ^]^ For example, segmented filamentous bacteria promote intestinal α1,2‐fucosylation by stimulating IL‐22 production from type 3 innate lymphoid cells (ILC3s), thereby protecting the intestine against *Salmonella typhimurium* infection.^[^ ^9^ ^]^ Similarly, *Bacteroides fragilis* restores α1,2‐fucosylation in bacteria‐depleted mice, facilitating recovery from dextran sulfate sodium (DSS)‐induced mucosal injury.^[^ ^10^ ^]^ Emerging evidence shows that microbiota‐derived metabolites induce host epigenetic modifications, influencing the expression and post‐translational modifications of proteins.^[^ ^11^, ^12^ ^]^ Notably, *B. thetaiotaomicron* strains lacking the ability to utilize *L*‐fucose fail to induce intestinal α1,2‐fucosylation, emphasizing the link between microbial fucose metabolism and host glycosylation.^[^ ^8^ ^]^ However, the molecular mechanisms through which probiotic‐derived metabolites regulate intestinal α1,2‐fucosylation remain poorly understood.

In addition to conventional drugs like 5‐aminosalicylates, biological therapies including probiotics are increasingly employed in the treatment of colitis.^[^ ^13^, ^14^ ^]^ Among probiotics, the mucus‐colonizing commensal *Akkermansia muciniphila* (*A. muciniphila*) has emerged as a promising probiotic candidate due to its anti‐inflammatory effects through active components including Amuc_1100, extracellular vesicles, and short‐chain fatty acids.^[^ ^15^, ^16^, ^17^, ^18^ ^]^ Similar to fucose‐utilizing strains of *B. thetaiotaomicron* that induce intestinal α1,2‐fucosylation, *A. muciniphila* also metabolizes fucose.^[^ ^8^, ^19^ ^]^ Therefore, we hypothesize that *A. muciniphila* may modulate intestinal fucosylation, contributing to its protective effect against colitis.

In this study, we assessed the intestinal fucosylation‐inducing capacity and its correlation with colitis‐ameliorating effects across four *A. muciniphila* strains. The results demonstrated that *A. muciniphila* alleviated colitis by enhancing intestinal α1,2‐fucosylation. We further identified its derivative, N‐acetylspermidine, as a key molecule responsible for upregulating α1,2‐fucosylation through the *HDAC2*‐*C1GALT1C1* axis. Enhanced α1,2‐fucosylation promoted the membrane localization of ZO‐1 and ZO‐2, crucial components of the gut barrier, while simultaneously suppressing C3 secretion, a factor implicated in inflammation. Together, these findings elucidate the mechanisms through which *A. muciniphila* maintains gut homeostasis by upregulating epithelial α1,2‐fucosylation, and underscore the potential of *A. muciniphila* and its derivative, N‐acetylspermidine, as promising candidates for developing biological therapies for colitis.

## 2 Result

### 2.1 *A. muciniphila* Alleviates Colitis by Enhancing Colonic α1,2‐Fucosylation

**Figure 1:**
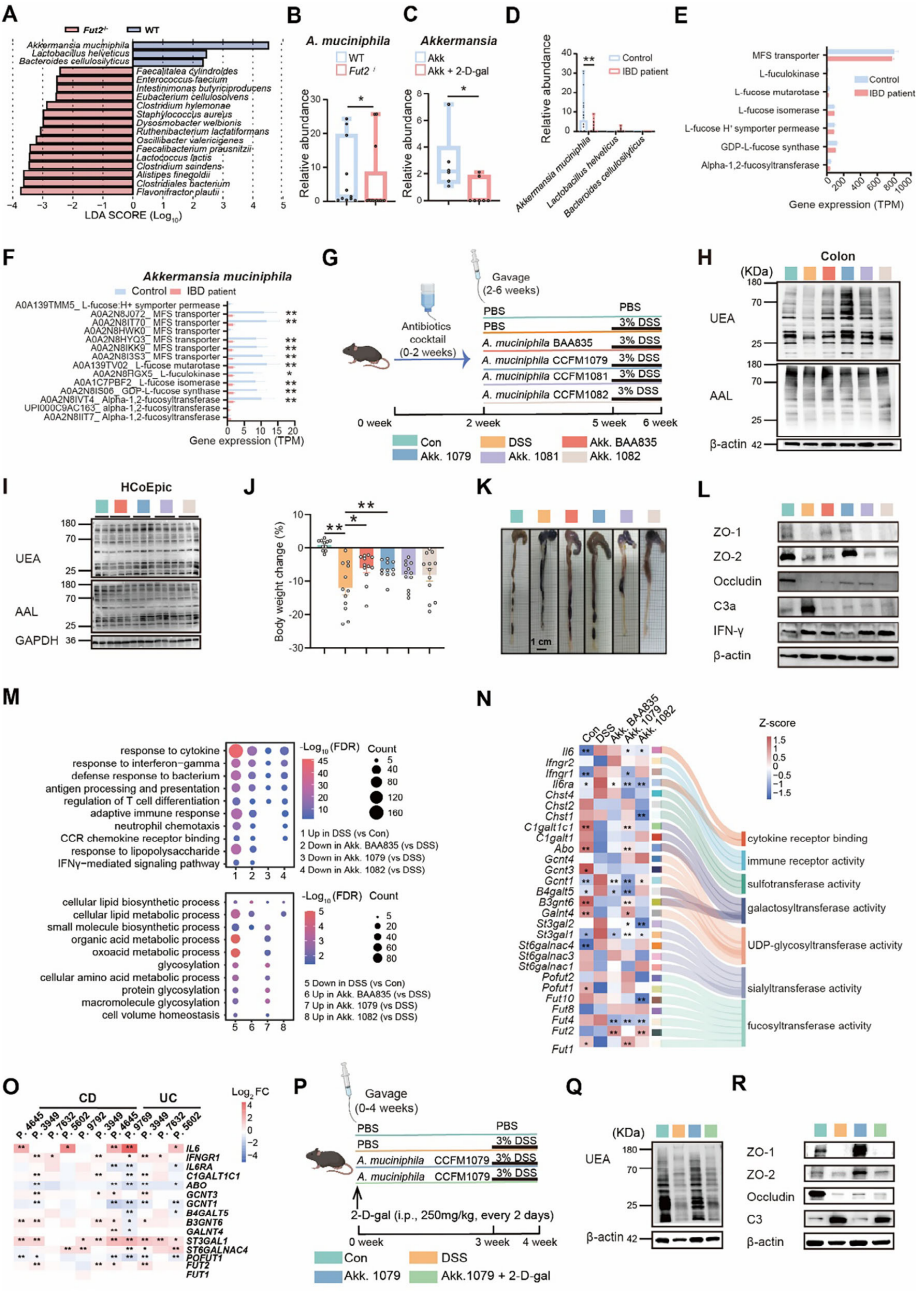
*A. muciniphila*‐induced intestinal α1, 2‐fucosylation alleviates colitis. A,B) Linear discriminant analysis (LDA) effect size (LEfSe) analysis (A) of gut microbiota and relative abundance of *A. muciniphila* (B) in Wild‐type (WT) and *Fut2* ^‐/‐^ mice based on public data (PRJNA614498). C) Relative abundance of the *Akkermansia* genus in the cecum of colitis mice gavaged with *A. muciniphila* and those receiving intraperitoneal injections of 2‐deoxy‐*D*‐galactose (2‐D‐gal, α1,2‐fucosylation inhibitor), *n* = 6–7. D) Relative abundance of *A. muciniphila* in IBD patients (PRJNA385949). E,F) Expression of genes involved in fucose synthesis and salvage pathway in whole gut microbiota (E) and those specifically from *A. muciniphila* in IBD patients (F) (PRJNA385949). G) Experiment design for colitis mice undergoing 2‐week antibiotic cocktail treatment followed by 4‐week oral gavage of 1 × 10^9^ CFU *A. muciniphila*, *n* = 12. H) Lectin blotting analysis of colonic α1, 2‐fucosylation (UEA) and α1, 6‐fucosylation (AAL) levels in the colonic tissues of control mice, colitic mice, and those gavage‐fed with *A. muciniphila*. I) Lectin blotting analysis of colonic α1, 2‐fucosylation and α1, 6‐fucosylation in HcoEpiC co‐cultured with *A. muciniphila* at a multiplicity of infection (MOI) of 100 for 4 h. J–L) Changes of body weight, *n* = 12 (J), representative colon images (K), and immunoblotting analysis of ZO‐1, ZO‐2, Occludin, C3a, and IFN‐γ (L) in colons of *A. muciniphila‐treated* colitis mcie. M,N) Bubble chart showing representative biological processes (M) and heatmap displaying representative molecular functions (N) of differentially expressed genes in colons of control mice, colitis mice, and those gavaged with *A. muciniphila*. FDR, False Discovery Rate. O) Heatmap of expression fold change in genes related to glycosylation and inflammation across intestinal tissues in IBD patients and control individuals (Public data, P. 4645, PRJEB24645; P. 3949, PRJNA483949; P. 7632, PRJNA507632; P. 5602, PRJNA985602; P. 9792, PRJNA659792; P. 9769, PRJNA429769). P) Experiment design for colitis mice gavaged with *A. muciniphila* CCFM1079, alongside intraperitoneal injections of 2‐deoxy‐*D*‐galactose, *n *= 8. Q,R) Lectin blotting analysis of colonic α1, 2‐fucosylation (Q) and immunoblotting analysis of ZO‐1, ZO‐2, Occludin, C3 (R) in colitis mice gavaged with *A. muciniphila* CCFM1079 and intraperitoneally injected with 2‐deoxy‐*D‐*galactose. Data are representative results of three independent experiments (H–L,P–R). Non‐normally distributed data is presented as Median (Interquartile Range, IQR) (B–D) and normally distributed data is presented as means ± SEM (E,F,J). Statistical analysis was conducted using one‐way analysis of variance (ANOVA), followed by Fisher's LSD tests with multiple comparisons adjustments (J) and Mann–Whitney *U*‐test (B–D). Statistical analysis of RNA‐seq data was performed using edgeR (O). *P < 0.05; **P < 0.01.

Impaired intestinal fucosylation in IBD highlights the therapeutic potential of targeting fucosylation pathways to restore mucosal homeostasis.^[^ ^20^, ^21^ ^]^ To explore the impact of α1,2‐fucosylation deficiency on the gut microbiota, we reanalyzed metagenomic sequencing data from fecal samples of *Fut2* ^‐/‐^ mice.^[^ ^22^ ^]^ Notably, the abundance of *A. muciniphila* was significantly reduced in *Fut2* ^‐/‐^ mice (**Figure** **1**A,B). Similarly, inhibition of α1,2‐fucosylation using 2‐deoxy*‐D*‐galactose in our study also led to a decline in *A. muciniphila* levels in the gut (Figure 1C). Analysis of public metagenomic sequencing data^[^ ^23^ ^]^ revealed that *A. muciniphila* levels and its fucose utilization and synthesis gene abundance were significantly diminished in IBD patients, despite unchanged overall microbial fucose metabolism gene abundance (Figure 1D–F). These findings suggest a potential regulatory function of *A. muciniphila* in epithelial fucosylation.

To investigate whether *A. muciniphila* influences intestinal fucosylation, four genomically distinct human‐derived strains were colonized in a colitis mouse model (Figure 1G; Figure S1A, Supporting Information). Before gavage, we administered an antibiotic cocktail to deplete gut microbiota, establishing a controlled low‐biomass environment essential for isolating *A. muciniphila*‐specific effects (Figure 1G). *A. muciniphila* reversed the colitis‐induced colonic α1,2‐fucosylation reduction (detected by Ulex Europaeus Agglutinin, UEA), within strain CCFM1079 showing the most significant effect (Figure 1H; Figure S1B, Supporting Information). In contrast, *A. muciniphila* did not affect the levels of intestinal α1,6‐fucosylation (detected by Aleuria Aurantia Lectin, AAL) (Figure 1H; Figure S1C, Supporting Information). Consistent with in vivo findings, human colonic epithelial cells (HcoEpiC) co‐cultured with *A. muciniphila* in vitro exhibited increased α1,2‐fucosylation levels without altering α1,6‐fucosylation levels, suggesting that *A. muciniphila* can selectively enhance intestinal α1,2‐fucosylation (Figure 1I; Figure S1D,E, Supporting Information). Furthermore, *A. muciniphila* CCFM1079 and BAA835 significantly alleviated DSS‐induced weight loss, colon shortening, histopathological damage, and tight junction disruption (Figure 1J–L; Figure S1F–K, Supporting Information). These strains downregulated the inflammatory markers C3a and IFN‐γ, and suppressed IL‐22, a cytokine pathologically associated with UC severity (Figure 1L; Figure S1L–N, Supporting Information). These results indicate that strains BAA835 and CCFM1079, which strengthen the gut barrier, may attenuate systemic inflammation in colitic mice. To further explore the systemic effects of *A. muciniphila*, we treated RAW264.7 cells with serum collected from colitis mice gavaged with these strains and performed RNA‐seq analysis. Serum from colitis mice elevated the expression of inflammation‐associated genes, such as *Il17c* and *Lcn2*, whereas serum from mice treated with *A. muciniphila* CCFM1079 reversed these effects (Figure S1O,P, Supporting Information). These findings demonstrate that *A. muciniphila* suppresses systemic inflammation.

We next investigated the impact of *A. muciniphila* on gene expression in the colon using RNA‐seq analysis. *A. muciniphila* significantly altered the transcriptional profiles of colitic mice, making them more similar to those of control mice (Figure S1Q–U, Table S1, Supporting Information). Gene Ontology (GO) analysis revealed that *A. muciniphila* reduced the expression of genes involved in inflammation‐related pathways (Figure 1M, upper). Additionally, the expression of genes involved in glycosylation was downregulated in colitis, which was restored by *A. muciniphila* CCFM1079 (Figure 1M, bottom). Among the 416 genes specifically upregulated by *A. muciniphila* CCFM1079, several were associated with fucosylation (e.g., *Galnt4*, *C1galt1c1*, *B3gnt6*, *Abo*), while none were linked to sialylation or sulfation (Figure 1N; Figure S1V, Supporting Information). Analysis of public RNA‐seq data from IBD patients^[^ ^24^, ^25^, ^26^, ^27^, ^28^, ^29^ ^]^ further revealed differential expression of fucosylation‐related genes (e.g., *ABO*, *FUT2*, *B3GNT6*, and *C1GALT1C1*) in comparison with control (Figure 1O).

Given that strain CCFM1079 exhibits the substantial colitis‐alleviating effect and the greatest capacity to upregulate colonic α1,2‐fucosylation, we hypothesized that α1,2‐fucosylation may be essential in *A. muciniphila*‐mediated colitis relief. Therefore, we assessed the impact of the α1,2‐fucosylation inhibitor 2‐deoxy‐*D*‐galactose on *A. muciniphila* CCFM1079‐induced colitis alleviation (Figure 1P). 2‐*D*eoxy*‐D*‐galactose significantly suppressed α1,2‐fucosylation levels (Figure 1Q; Figure S2A, Supporting Information). The inhibition impaired the *A. muciniphila* CCFM1079‐induced increases in body weight, colon length, and expression of ZO‐1, ZO‐2, and Occludin, as well as the reductions in complement C3 expression, histopathological scores, and IL‐22 expression (Figure 1R; Figure S2B–K, Supporting Information). Collectively, *A. muciniphila*‐induced intestinal α1,2‐fucosylation is crucial for its colitis‐alleviating effects.

### 2.2 *A. muciniphila* Modulates IBD‐Linked Alterations in α1,2‐Fucosylated Proteins Expression Patterns

**Figure 2:**
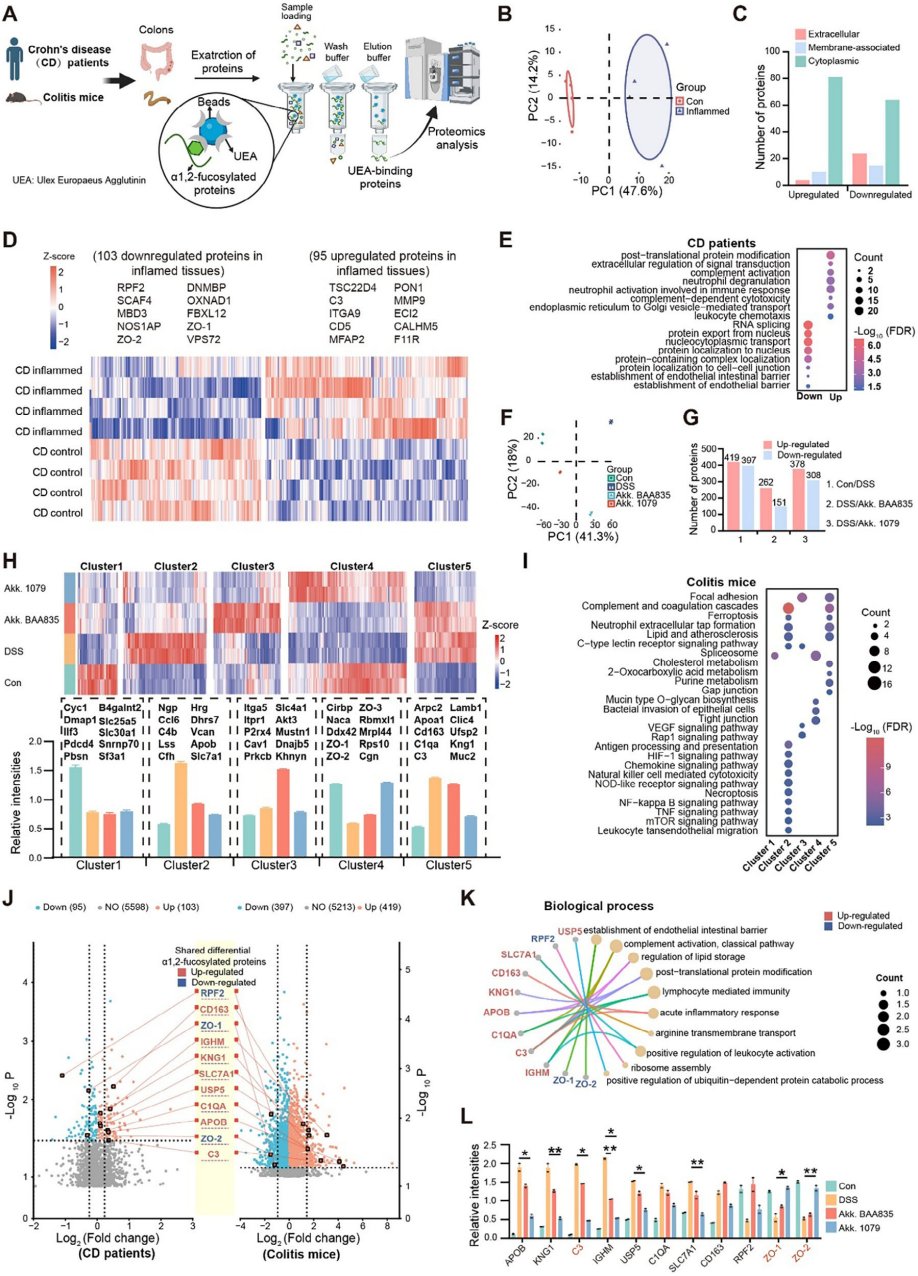
*A. muciniphila* modulates IBD‐linked alterations in α1,2‐fucosylated proteins expression patterns. A) Flow chart illustrating the enrichment of α1,2‐fucosylated proteins in colons of Crohn's disease (CD) patients and colitis mice using agarose‐bound Ulex europaeus agglutinin and detection using proteomics. B) Principal component analysis (PCA) of α1,2‐fucosylated proteins in control and inflamed colon tissues of CD patients, *n* = 4. C) The number of extracellular, membrane‐associated, and cytoplasmic proteins in upregulated and downregulated α1,2‐fucosylated proteins of inflamed colon tissues compared to control tissues. D) Heatmap showing differentially expressed α1,2‐fucosylated proteins between control and inflamed tissues of CD patients. E) Bubble plot showing representative biological processes of differentially expressed α1, 2‐fucosylated proteins in the colons of CD patients. F,G) PCA (F) and the number of (G) differentially expressed α1, 2‐fucosylated proteins of control mice, colitis mice, and those supplemented with *A. muciniphila* BAA835 and *A. muciniphila* CCFM1079, *n* = 2. H,I) Clustering analysis and relative intensities (H), and bubble chart showing representative KEGG pathways (I) of differentially expressed α1,2‐fucosylated proteins among control mice, colitic mice, and those administered with *A. muciniphila* BAA835 and *A. muciniphila* CCF1079. J) Double volcano plots showing the expression pattern of α1,2‐fucosylated proteins of colons from CD patients and colitis mice. K) Chord diagram showing the representative biological processes of shared differential α1,2‐fucosylated proteins in colons from CD patients and colitis mice. L) Relative intensities of shared differentially α1,2‐fucosylated proteins from CD patients and colitis mice. Data are representative results of two independent experiments (F–I). Error bars represent the SEM. Statistical analysis was conducted using the Standard coefficient of variation (*CV*) (L). *CV < 0.1; **CV < 0.01.

To characterize alterations in intestinal α1,2‐fucosylated protein patterns in inflamed gut tissues, we isolated these proteins from colon tissues of CD patients and colitis mice using agarose‐bound UEA followed by LC‐MS/MS analysis (**Figure** **2**A). In CD patients, α1,2‐fucosylated protein profiles exhibited distinct compositional differences between inflamed and non‐inflamed colonic tissues (Figure 2B). A total of 5796 α1,2‐fucosylated proteins were identified, with 198 showing differential expression (Figure 2C,D, Table S2, Supporting Information). Functionally, 103 upregulated proteins were primarily associated with inflammation, such as complement activation (Figure 2E; Figure S3A, Supporting Information). 95 downregulated proteins were linked to barrier maintenance, such as protein localization to the cell–cell junction (Figure 2E; Figure S3A, Supporting Information).

Consistent with observations in CD patients, colitis caused distinct remodeling in the composition of α1,2‐fucosylated proteins in mice (Figure 2F). LC‐MS/MS identified 6029 α1,2‐fucosylated proteins, with 816 differentially expressed between control and colitis mice (Figure 2G, Table S2, Supporting Information). The α1,2‐fucosylated protein profiles in *A. muciniphila* CCFM1079‐treated mice were significantly closer to controls than those in *A. muciniphila* BAA835‐treated mice (Figure 2H). Specifically, *A. muciniphila* CCFM1079 restored the reduced expression of certain α1,2‐fucosylated proteins in colitis mice, including membrane‐associated proteins (ZO‐1 and ZO‐2), which are critical for epithelial integrity maintenance (Figure 2H,I; Figure S3B, Supporting Information). Additionally, strain CCFM1079 reversed the elevated expression of α1,2‐fucosylated proteins involved in immune regulation, such as the secretory protein C3 (Figure 2H,I; Figure S3B, Supporting Information).

Integrated analysis of CD patients and colitis mice datasets identified eight upregulated α1,2‐fucosylated proteins (e.g., C3) in both CD patients and colitis mice that were related to immune responses (Figure 2J,K). Three downregulated proteins (e.g., ZO‐1, ZO‐2) were involved in intestinal barrier establishment (Figure 2J,K). Among these 11 proteins, *A. muciniphila* CCFM1079 most significantly reversed C3, ZO‐1, and ZO‐2 levels, suggesting them as critical mediators in its colitis‐alleviating effects (Figure 2L). Furthermore, public single‐cell sequencing data^[^ ^30^ ^]^ analysis revealed decreased *TJP1* (ZO‐1) and *TJP2* (ZO‐2) expression, alongside increased C3 expression, across multiple cell clusters in IBD (Figure S3C,D, Supporting Information).

### 2.3 *A. muciniphila*‐Derived *N*‐Acetylspermidine Mediates Colitis Alleviation

**Figure 3:**
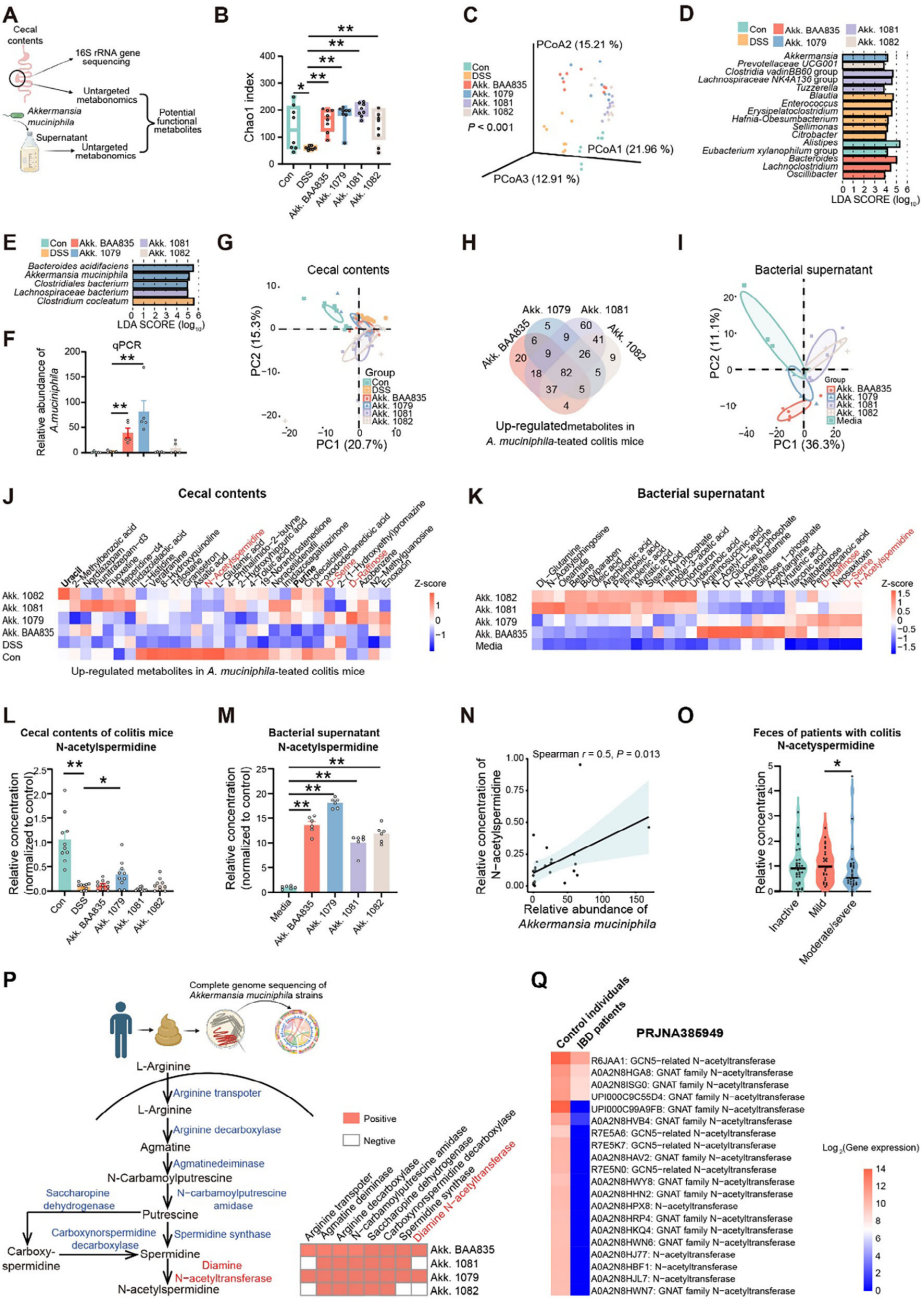
*A. muciniphila* alters gut microbiota composition and metabolism of colitis mice. A) Study design of 16S rRNA gene sequencing and metabolomics based on cecal contents of *A. muciniphila‐*gavaged colitis mice and supernatants of *A. muciniphila*. B,C) Chao 1 index (B) and principal coordinate analysis (C) of cecal microbiota based on 16S rRNA gene sequencing from control mice, colitic mice, and those gavaged with *A. muciniphila*, *n* = 8. D,E) LEfSe analysis showed the differential genera (D) and species (E) in cecal microbiota. F) Relative abundance of cecal *A. muciniphila* detected by qPCR, *n* = 5. G) PCA based on untargeted metabolomics data from cecal contents of mice, *n* = 10. H) Venn diagram showing the up‐regulated cecal metabolites of colitis mice gavaged with *A. muciniphila* compared with that of colitis mice. I) PCA of untargeted metabolomics of the supernatants from four *A. muciniphila* strains, *n* = 6. J,K) Heatmap showing the relative concentrations of upregulated cecal metabolites in *A. muciniphila‐*treated colitis mice (J) and metabolites in the supernatants of *A. muciniphila* (K). L) Relative concentrations (normalized to the control) of N‐acetylspermidine in the cecum of control mice, colitic mice, and those gavage‐fed with *A. muciniphila* strains, *n* = 10. M) Relative concentrations (normalized to the media) of N‐acetylspermidine in the supernatants of four *A. muciniphila* strains, *n* = 6. N) Dot plot depicting the correlation between relative concentration of N‐acetylspermidine and relative abundance of *A. muciniphila* (detected by qPCR) in cecal metabolites. O) Relative concentrations of N‐acetylspermidine in patients with varying severities of UC (Inactive, *n* = 35; mild, *n* = 27; moderate/severe, *n* = 30) (data from NIH Common Fund's National Metabolomics Data Repository website, PR001596). P) N‐acetylspermidine synthesis pathway and the presence of these genes in the complete genomes of *A. muciniphila* strains. Q) The expression of genes encoding N‐acetyltransferase from *A. muciniphila* in the feces of IBD patients and control individuals (PRJNA385949). Data are representative results of three independent experiments (F,G,I–M). Non‐normally distributed data is presented as Median (Interquartile Range, IQR) (B) and normally distributed data is presented as means ± SEM (F,L,M). Statistical analysis was performed using the Kruskal–Wallis tests followed by uncorrected Dunn's test with multiple comparison adjustments (B) and one‐way ANOVA followed by Fisher's LSD tests with for multiple comparison adjustments (F,L,M,O) and LEfSe analysis (D,E). *P < 0.05; **P < 0.01.

To assess the impact of *A. muciniphila* on the gut microbiota structure of colitis mice, we conducted a 16S rRNA gene sequencing analysis on cecal contents (**Figure** **3**A). *A. muciniphila* intervention increased the Chao 1 index and observed features, and restructured gut microbiota composition of colitis mice (Figure 3B,C; Figure S4A, Supporting Information). Specifically, linear discriminant analysis effect size (LEfSe) analysis revealed an increased abundance of *Akkermansia* and *A. muciniphila* in mice receiving *A. muciniphila* CCFM1079 supplementation (Figure 3D,E; Figure S4B, Supporting Information). The qPCR analysis confirmed a 34‐fold increase in *A. muciniphila* abundance in colitic mice administered with *A. muciniphila* CCFM1079, demonstrating its superior intestinal colonization capacity (Figure 3F). Additionally, *A. muciniphila* CCFM1079 promoted the growth of beneficial bacteria such as *Faecalibaculum* and *Lachnoclostridium*, while reducing pathogenic bacteria *Escherichia/Shigella* (Figure S4B, Supporting Information). Network analysis showed a positive correlation between *Akkermansia* and beneficial bacteria, including *Faecalibaculum* (Figure S4C, Supporting Information).

To identify *A. muciniphila‐*derived metabolites governing fucosylation regulation and colitis alleviation, we conducted untargeted metabolomics on the cecal contents of colitis mice and *A. muciniphila* culture supernatants (Figure 3A). *A. muciniphila* influenced the composition of cecal metabolites, with different strains upregulating unique metabolites (Figure 3G,H). Notably, the metabolic profiles of high‐efficacy colitis‐alleviating strains (BAA835 and CCFM1079) differed from the less efficacy strains (CCFM1081 and CCFM1082) (Figure 3I). Among the metabolites of *A. muciniphila*, *N*‐acetylspermidine and D‐serine—significantly depleted in colitic mice—were restored by *A. muciniphila* CCFM1079 treatment (Figure 3J–M; Figure S4D, Supporting Information). Correlation analysis identified a positive association between *A. muciniphila* abundance and *N*‐acetylspermidine levels, suggesting *A. muciniphila* CCFM1079 as a primary source of *N*‐acetylspermidine in the gut of treated colitis mice (Figure 3N). Additionally, analysis based on publicly available untargeted metabolomics data^[^ ^31^ ^]^ further validated significantly reduced fecal *N*‐acetylspermidine levels in patients with moderate or severe UC (Figure 3O).

Given that divergent metabolite production may underlie genomic distinctions, we sequenced the complete genomes of the three strains of *A. muciniphila* and conducted a comparative genomic analysis based on the three genomes and a genome of strain BAA835 from NCBI (Figure 3P; Figure S4E, Table S3, Supporting Information). *A. muciniphila* CCFM1079 possesses 135 unique genes, notably a PFAM cytochrome c biogenesis protein, which is associated with colonization (Figure S4F, Supporting Information). Both *A. muciniphila* BAA835 and CCFM1079 encoded a complete *N*‐acetylspermidine biosynthesis pathway, while strains CCFM1081 and CCFM1082 lacked the essential genes required for converting spermidine to *N*‐acetylspermidine (Figure 3P). Analysis of public metagenomic data of stool samples^[^ ^23^ ^]^ indicated a significant reduction in genes encoding *N‐*acetyltransferase of *A. muciniphila* in IBD patients, suggesting diminished *N*‐acetylspermidine biosynthesis capacity in IBD (Figure 3Q).

### 2.4 *N*‐Acetylspermidine Enhances the Gut Barrier by Increasing the α1,2‐Fucosylation Levels

**Figure 4:**
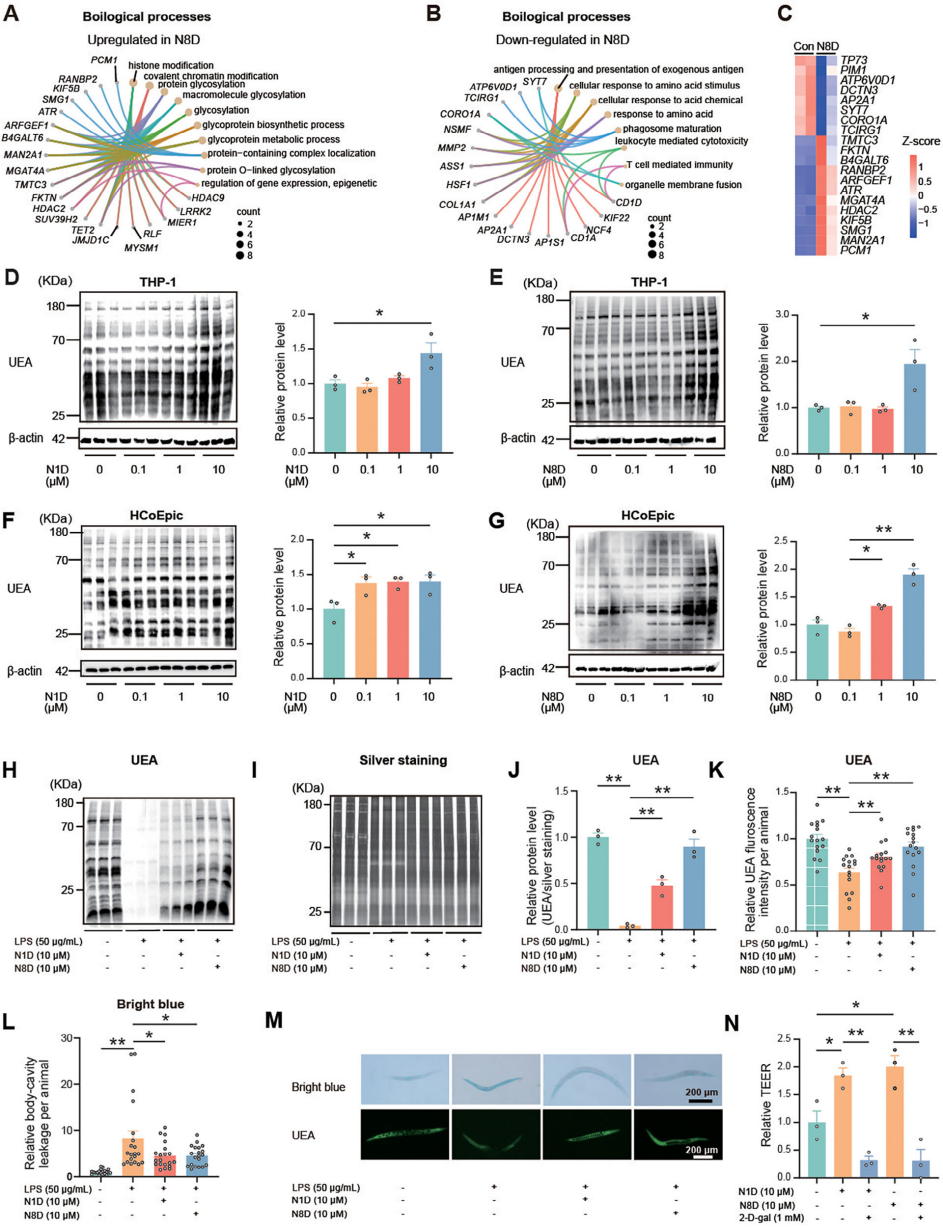
*A. muciniphila*‐derived N‐acetylspermidine strengthens the gut barrier by enhancing α1, 2‐fucosylation in vivo and in vitro. A,B) Chord diagram presenting representative biological processes of upregulated (A) and downregulated (B) genes in THP‐1 cells treated with N8‐acetyspermidine (N8D) for 12 h, *n* = 2. C) Heatmap of the expression of differentially expressed genes in THP‐1 cells treated with N8D for 12 h. D,E) Lectin blotting analysis of α1, 2‐fucosylation in THP‐1 cells treated with N1‐acetylspermidine (N1D) (D) and N8D (E) for 24 h. F,G) Lectin blotting analysis of α1, 2‐fucosylation in HcoEpiC treated with N1D (F) and N8D (G) for 24 h. H–M) UEA blotting (H), silver staining (I), relative α1, 2‐fucosylation levels (J), relative UEA fluorescence intensities (K) and brilliant blue body‐cavity leakages (L), representative images of brilliant blue staining (upper) and UEA staining (bottom) (M) of *C. elegans* treated with N1D and N8D for 48 h, followed by co‐treatment with lipopolysaccharide (LPS) for additional 24 h, *n* = 30. N) TEER values of HcoEpiC treated with N1D and N8D, in combination with 2‐*D‐*gal for 48 h, *n* = 3. Data are representative results of three independent experiments (D–N). Error bars represent the SEM. Statistical analysis was conducted using one‐way ANOVA followed by Fisher's LSD tests with multiple comparisons (D–G,J–L,N). *P < 0.05; **P < 0.01.

To explore the biological functions of *N*‐acetylspermidine, we performed RNA‐seq analysis on leukemia monocyte cells (THP‐1) treated with N8‐acetylspermidine (N8D). Transcriptomic profiling of N8D‐exposed THP‐1 cells uncovered profound gene expression reprogramming, validated by coordinated PCA and volcano plot signatures (Figure S5A,B, Supporting Information). Specifically, N8D downregulated genes related to T cell‐mediated immunity, while upregulating histone modification and glycosylation genes, suggesting its potential role in regulating α1,2‐fucosylation (**Figure** **4**A–C, Table S4, Supporting Information). Lectin blotting analysis demonstrated that both N1‐acetylspermidine (N1D) and N8D elevated the α1,2‐fucosylation levels in THP‐1 and HCoEpic (Figure 4D–G).

To further evaluate the effect of *N*‐acetylspermidine on α1,2‐fucosylation and colitis in vivo, we established a colitis model in *Caenorhabditis elegans* (*C. elegans*) by inducing gut barrier injury with lipopolysaccharide (LPS). Consistent with the in vitro findings, *N*‐acetylspermidine rescued the LPS‐caused substantial decrease in α1,2‐fucosylation levels in *C. elegans* (Figure 4H–K,M). Additionally, *N*‐acetylspermidine ameliorated LPS‐induced intestinal injury, as evidenced by a reduced bright blue body‐cavity leakage (Figure 4L,M). Moreover, the α1,2‐fucosylation inhibition abrogated the N‐acetylspermidine‐induced enhancement in transepithelial electrical resistance (TEER) of HcoEpiC (Figure 4N).

### 2.5 *A. muciniphila*‐Derived N‐Acetylspermidine Enhances α1,2‐Fucosylation via the *HDAC2*‐*C1GALT1C1* Axis

**Figure 5:**
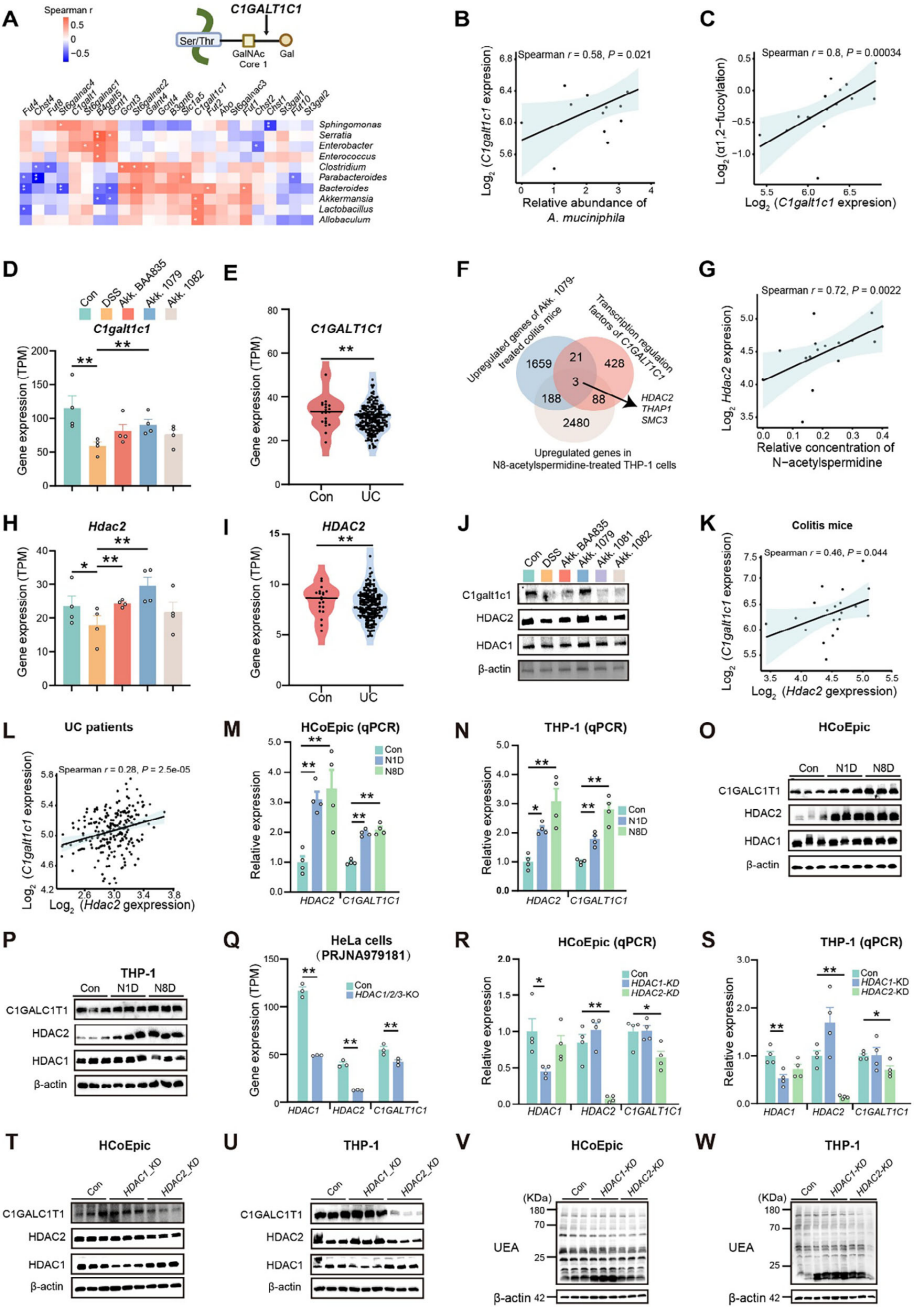
*A. muciniphila*‐derived N‐acetylspermidine increases the expression of *C1GALT1C1* by upregulating *HDAC2* expression. A) Heatmap of Spearman correlation analysis depicting the relationship between the expression of genes involved in glycosylation and the abundance of genera in the intestines of colitis mice, and a schematic diagram of the function of *C1GALT1C1*. B,C) Dot plot depicting the correlation between *C1galt1c1* expression and *A. muciniphila* abundance (B) and α1,2‐fucosylation levels (C) in the intestines of colitis mice. D) Gene expression of *C1galt1c1* in colons of *A. muciniphila*‐treated colitis mice, *n* = 4. E) Violin plot showing the gene expression of *C1GALT1C1* in rectal mucosal tissues of control individuals and patients with ulcerative colitis (UC) (PRJNA429769), con, *n* = 20; UC, *n* = 202. F) Venn diagram showing the sharing upregulated genes in colons of colitis mice administered with *A. muciniphila* CCFM1079, predicted transcription factors of *C1GALT1C1*, and upregulated genes in N8‐acetylspermidine‐treated THP‐1 cells. G) Dot plot depicting the correlation between the N‐ acetylspermidine concentrations and *Hdac2* expression in *A. muciniphila*‐treated colitis mice. H) Gene expression of *Hdac2* in colons of *A. muciniphila*‐treated colitis mice, *n* = 4. I) Violin plot showing the gene expression of *HDAC2* in rectal mucosal tissues of control individuals and UC patients (PRJNA429769), con, *n* = 20; UC, *n* = 202. J) Immunoblot analysis of HDAC1, HDAC2, C1galt1c1 in colons of colitis mice gavaged with *A. muciniphila*. K) Dot plot depicting the correlation between the gene expression of *C1galt1c1* and *Hdac2* in colitis mice and those gavaged with *A. muciniphila*. L) Dot plot showing the correlation between the *C1GALT1C1* and *HDAC2* expression in rectal mucosal tissues of UC patients and control individuals (PRJNA429769). M,N) Relative expression (detected by qPCR) of *HDAC2* and *C1GALT1C1* in HcoEpiC (M) and THP‐1 cells (N) treated with N1D and N8D for 12 h, *n* = 4. O,P) Immunoblot analysis of HDAC1, HDAC2, C1GALT1C1 in HcoEpiC (O) and THP‐1 cells (P) treated with N1D and N8D for 24 h. Q) Gene expression of *HDAC1*, *HDAC2* and *C1GALT1C1* of HDAC1/2/3‐knocknout HeLa cells (PRJNA979181), *n* = 3. R,S) Relative expression (detected by qPCR) of *HDAC1*, *HDAC2*, and *C1GALT1C1* in HcoEpiC (R) and THP‐1 cells (S) treated with siRNA targeted to *HDAC1* and *HDAC2* for 24 h, *n* = 4. T,U) Immunoblot analysis of HDAC1, HDAC2, C1GALT1C1 in HcoEpiC (T) and THP‐1 cells (U) treated with siRNA targeted to *HDAC1* and *HDAC2* for 48 h, *n* = 3. V,W) Lectin blot analysis of α1,2‐fucosylation in HcoEpiC (V) and THP‐1 cells (W) treated with siRNA targeted to *HDAC1* and *HDAC2* for 48 h, *n* = 3. Data are representative results of three independent experiments (J,M–R,T–W). Error bars represent the SEM. Statistical analysis was performed using one‐way ANOVA followed by Fisher's LSD tests with multiple comparisons adjustments (D,H,M,N,R,S) and unpaired Student's *t*‐test (E,I,Q). *P < 0.05; **P < 0.01.

Both N‐acetylspermidine and *A. muciniphila* CCFM1079 significantly enhanced α1,2‐fucosylation without increasing IL‐22 or *Fut2* expression (Figures 1H and 4D–M; Figures S1N,S7A, Supporting Information), suggesting the existence of alternative mechanisms of α1,2‐fucosylation regulation in addition to the IL‐22‐*Fut2* pathway. Correlation analysis revealed that the cecal abundance of *Akkermansia* and *A. muciniphila* positively correlated with colonic *C1galt1c1* expression (**Figure** **5**A,B). *C1galt1c1* (core 1 β1,3‐galactosyltransferase‐specific chaperone 1), also known as *Cosmc*, catalyzes the synthesis of the core 1 structure, which serves as a substrate for α1,2‐fucosylation (Figure 5A). The *C1galt1c1* expression also positively correlated with colonic α1,2‐fucosylation levels, suggesting its essential role in α1,2‐fucosylation regulation (Figure 5C). Furthermore, *A. muciniphila* CCFM1079, which exhibited superior colitis‐alleviating and α1,2‐fucosylation‐enhancing effects, restored *C1galt1c1* expression in colitis mice (Figure 5D). Analysis of public RNA‐seq data^[^ ^29^ ^]^ further demonstrated a decrease in *C1GALT1C1* expression in the rectal mucosal tissues from UC patients (Figure 5E).

Of the 540 predicted candidate regulatory genes, only *HDAC2*, *THAP1*, and *SMC3* were upregulated by *A. muciniphila* CCFM1079 and N8‐acetylspermidine (Figure 5F). Notably, only *HDAC2* (histone deacetylase 2) exhibited a positive correlation with both cecal N‐acetylspermidine levels and *A. muciniphila* abundance (Figure 5G; Figure S6A,B, Supporting Information). Consistently with its effect on *C1galt1c1*, *A. muciniphila* CCFM1079 increased *Hdac2* expression in colitis mice, and *HDAC2* expression was also decreased in UC patients (Figure 5H,I). Immunoblotting analysis confirmed elevated HDAC2 and C1galt1c1 expression levels in *A. muciniphila* CCFM1079‐treated colitis mice (Figure 5J; Figure S6C–E, Supporting Information). Additionally, correlation analysis confirmed a positive association between *HDAC2* and *C1GALT1C1* transcription levels in colitis mice and multiple human intestinal tissues, indicating *HDAC2*‐mediated transcriptional regulation of *C1GALT1C1* (Figure 5K–L; Figure S6F–I, Supporting Information).

In vitro, N‐acetylspermidine increased *HDAC2* and *C1GALT1C1* transcription and protein levels in HcoEpiC and THP‐1 cells (Figure 5M–P; Figure S6J–K, Supporting Information). Analysis of public RNA‐seq data^[^ ^32^ ^]^ showed that *HDAC1/2/3* knockout reduced *C1GALT1C1* expression in HeLa cells (Figure 5Q). Furthermore, knockdown of *HDAC2* decreased *C1GALT1C* transcription and protein expression, as well as α1,2‐fucosylation levels, in THP‐1 and HcoEpiC (Figure 5R–W; Figure S6L–O, Supporting Information).

Together, these findings indicate that *A. muciniphila‐*derived N‐acetylspermidine enhanced α1,2‐fucosylation by upregulating *HDAC2*, which subsequently promotes *C1GALT1C1* expression.

### 2.6 N‐Acetylspermidine Upregulates *C1GALT1C1* Expression via *PIM1* Inhibition and Subsequent *HDAC2*‐Mediated Reduction in *TP73* Chromatin Accessibility

**Figure 6:**
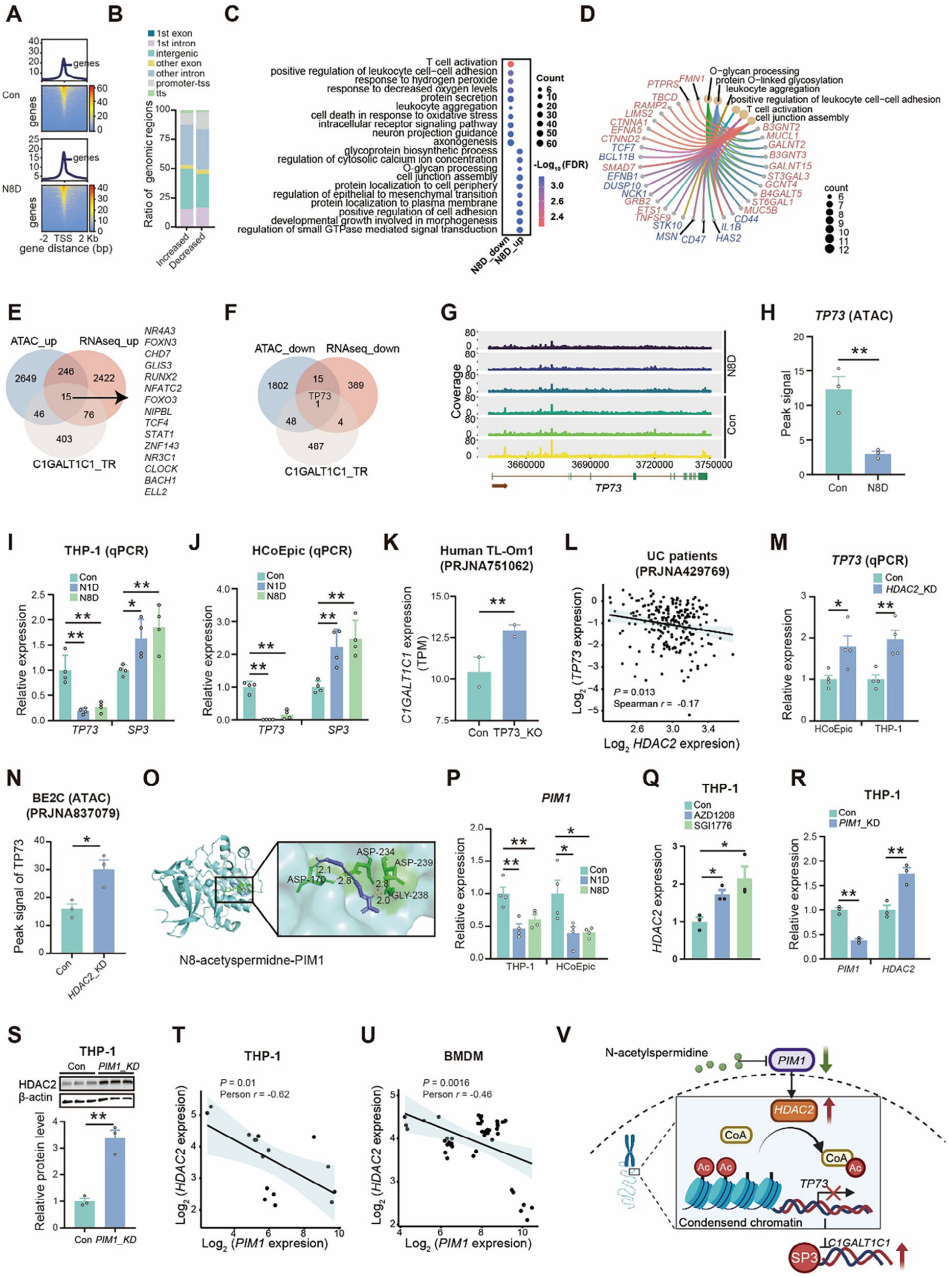
N‐acetylspermidine upregulates *C1GALT1C1* expression via *PIM1* inhibition and subsequent HDAC2‐miediated reduction in *TP73* chromatin accessibility. A) Nucleosome‐free regions (NFR) peak signaling distribution based on ATAC‐seq data of THP‐1 cells treated with N8D for 12 h, *n* = 3. B) The ratio of genomic functional regions in NFR peaks with differential signals in THP‐1 cells treated with N8D for 12 h. C,D) Bubble chart (C) and chord diagram (D) presenting representative biological processes of genes with differential NFR signals in THP‐1 cells treated with N8D for 12 h. E,F) Venn diagram showing the overlap between upregulated genes in RNA‐seq, genes with increased NFR signals in ATAC‐seq, and predicted transcriptional regulators of *C1GALT1C1* (E), as well as the overlap between downregulated genes in RNA‐seq, genes with NFR decreased signals in ATAC‐seq, and *C1GALT1C1* regulators (F) in N8D‐treated THP‐1 cells. G,H) ATAC‐seq genomic binding profiles (G) and the levels of NFR signals (H) at the *TP73* locus, *n* = 3. I,J) Relative expression (detected by qPCR) of *TP73* and *SP3* in THP‐1 cells (I) and HCoEpic (J) treated with N1D and N8D for 12 h. K) Gene expression levels of *CAGALT1C1* in *TP73*‐knockout Human TL‐Om1 cells (PRJNA751062). L) Dot plot showing the correlation between the gene expression of *HDAC2* and *TP73* in rectal mucosal tissues of UC patients and control individuals (PRJNA429769). M) Relative expression (detected by qPCR) of *TP73* in *HDAC2*‐knockdown THP‐1 cells and HCoEpic. N) The levels of NFR peak signal at the *TP73* locus of *HDAC2*‐knockout BE2C cells based on ATAC‐seq analysis (PRJNA837079). O) 3D structure illustrating interaction modes of N8‐acetylspermidine and PIM1 based on molecular docking analysis. P) Relative expression (detected by qPCR) of *PIM1* in THP‐1 cells and HCoEpic treated with N1D and N8D for 12 h. Q) Relative expression (detected by qPCR) of *HDAC2* in THP‐1 cells treated with PIM1 inhibitors AZD1208 and SGI1776 for 12 h, *n* = 3. R) Relative expression (detected by qPCR) of *PIM1* and *HDAC2* in THP‐1 cells treated with siRNA targeted to *PIM1*, *n* = 3. S) Immunoblot analysis of HDAC2 in THP‐1 cells treated with siRNA targeted to *PIM1*, *n* = 3. T) Dot plot showing the Pearson correlation between the gene expression of *PIM1* and *HDAC2* in THP‐1 cells (based on RNA‐seq data of N8D‐treated THP‐1 cells in this study and public data of THP‐1 cells form NCBI with accession numbers PRJNA394134, PRJNA858047, and PRJNA632845). U) Dot plot showing the pearson correlation between the gene expression of *PIM1* and *HDAC2* in BMDMs (data from NCBI with accession numbers PRJNA520989, PRJNA728581, PRJNA609623, and PRJNA385311). V) Mechanism diagram showing how N‐acetylspermidine upregulates *HDAC2* via *PIM1* inhibition, which decreases the chromatin accessibility of *TP73*, potentially inhibiting the SP3 expression and its binding to the *C1GALT1C1* enhancer, ultimately upregulating *C1GALT1C1*. Data are representative results of three independent experiments (I,J,M,P–S). Error bars represent the SEM. Statistical analysis was conducted using unpaired Student's *t*‐test (H,K,M,N,R,S) and one‐way ANOVA followed by Fisher's LSD tests with multiple comparisons adjustments (I,J,P,Q). *P < 0.05; **P < 0.01.

HDAC2 is generally known to promote chromatin condensation, reducing gene expression.^[^ ^33^ ^]^ However, the positive correlation between *HDAC2* and *C1GALT1C1* suggested that additional regulatory factors may influence *C1GALT1C1* expression (Figure 5D,H,K,L; Figures S6F–I,S7A, Supporting Information), which led us to hypothesize that N‐acetylspermidine may modulate chromatin accessibility. Assays for transposase‐accessible chromatin using sequencing (ATAC‐seq) showed that N8D significantly affected the chromatin accessibility of THP‐1 cells (**Figure** **6**A; Figure S7B, Supporting Information). The differential accessible genomic regions were enriched in intergenic, intronic, and promoter‐transcription start site (TSS) regions (Figure 6B). Among 1,011 nucleosome‐free regions (NFRs) with altered accessibility, N8D significantly increased chromatin openness at 590 NFRs, including loci within O‐glycosylation genes (Figure 6C,D; Figure S7A–D, Table S5, Supporting Information). Among *C1GALT1C1* transcriptional regulators, N8D upregulated transcription and chromatin accessibility in 15 genes, while uniquely suppressing *TP73* (Tumor Protein P73) expression and chromatin accessibility (Figure 6E–H).

*TP73* reduces gene expression by competitively inhibiting Sp3 binding at core promoters, including that of *C1GALT1C1*.^[^ ^34^, ^35^ ^]^ qPCR analysis confirmed that N‐acetylspermidine significantly downregulated *TP73* expression, while concomitantly upregulating *SP3* in THP‐1 cells and HCoEpic (Figure 6I,J). Reanalysis of public RNA‐seq data^[^ ^36^ ^]^ revealed that *TP73* knockout increased *C1GALT1C1* expression in Human TL‐Om1 cells (Figure 6K). Correlation analysis further revealed that *HDAC2* expression negatively correlated with *TP73* expression in UC patients (Figure 6L). Consistently, *HDAC2* knockdown promoted *TP73* transcription in HCoEpic and THP‐1 cells (Figure 6M). Furthermore, public ATAC data reanalysis^[^ ^37^ ^]^ showed that *HDAC2* knockdown increased chromatin accessibility at the *TP73* locus in human neuroblastoma cells (BE2C) (Figure 6N). Collectively, these findings suggest that *HDAC2* upregulates *C1GALT1C1* by reducing chromatin accessibility at the *TP73* locus.

Since HDAC2 primarily functions in the cell nucleus, it is plausible that additional regulatory factors may interact with HDAC2 in response to N‐acetylspermidine. PIM1, known to downregulate *HDAC2*, could act as a negative regulator of *HDAC2*.^[^ ^38^ ^]^ Therefore, we propose that N‐acetylspermidine may regulate the expression of *HDAC2* by binding to PIM1. Molecular docking analysis showed a lower binding free energy between N8‐acetylspermidine and PIM1 (−7.84 kcal mol^−1^) compared to HDAC2 (−4.36 kcal mol^−1^), suggesting a stronger binding affinity for PIM1 (Figure 6O, Table S6, Supporting Information). qPCR and RNA‐seq further confirmed that N‐acetylspermidine downregulated *PIM1* expression (Figure 6P; Figure S7A, Supporting Information). Additionally, PIM1 inhibition using specific antagonists (AZD1208 and SGI1776) upregulated both the transcription and protein expression levels of HDAC2 in THP‐1 cells (Figure 6Q; Figure S8A,B, Supporting Information). Likewise, PIM1 knockdown increased the *HDAC2* transcriptional and protein levels in THP‐1 cells, as well as *HDAC2* transcription levels in HCoEpic (Figure 6R,S, Figure S8C,D, Supporting Information). Our integrated analysis of multiple public datasets^[^ ^39^, ^40^, ^41^, ^42^, ^43^, ^44^, ^45^ ^]^ also revealed a negative correlation between *HDAC2* and *PIM1* expression in THP‐1 cells, bone marrow‐derived macrophages (BMDMs), and human esophagus mucosa (Figure 6T,U; Figure S8E, Supporting Information). Furthermore, reanalysis of public RNA‐seq data^[^ ^46^ ^]^ indicated that *Pim1* knockout elevated *Hdac2* and *C1galt1c1* expression in BMDMs, and higher *C1galt1c1* expression was observed in intestinal epithelial cells (IECs) from *Pim1*‐deficient mice compared to wild‐type mice (Figure S8F,G, Supporting Information).

In summary, these findings suggest that N‐acetylspermidine upregulates *HDAC2* expression, through PIM1 inhibition, thereby reducing chromatin accessibility at the *TP73* locus and ultimately enhancing *C1GALT1C1* expression (Figure 6V).

### 2.7 N‐Acetylspermidine‐Induced α1,2‐Fucosylation Suppresses C3 Secretion and Promoted the Membrane Location of ZO‐1 and ZO‐2

**Figure 7:**
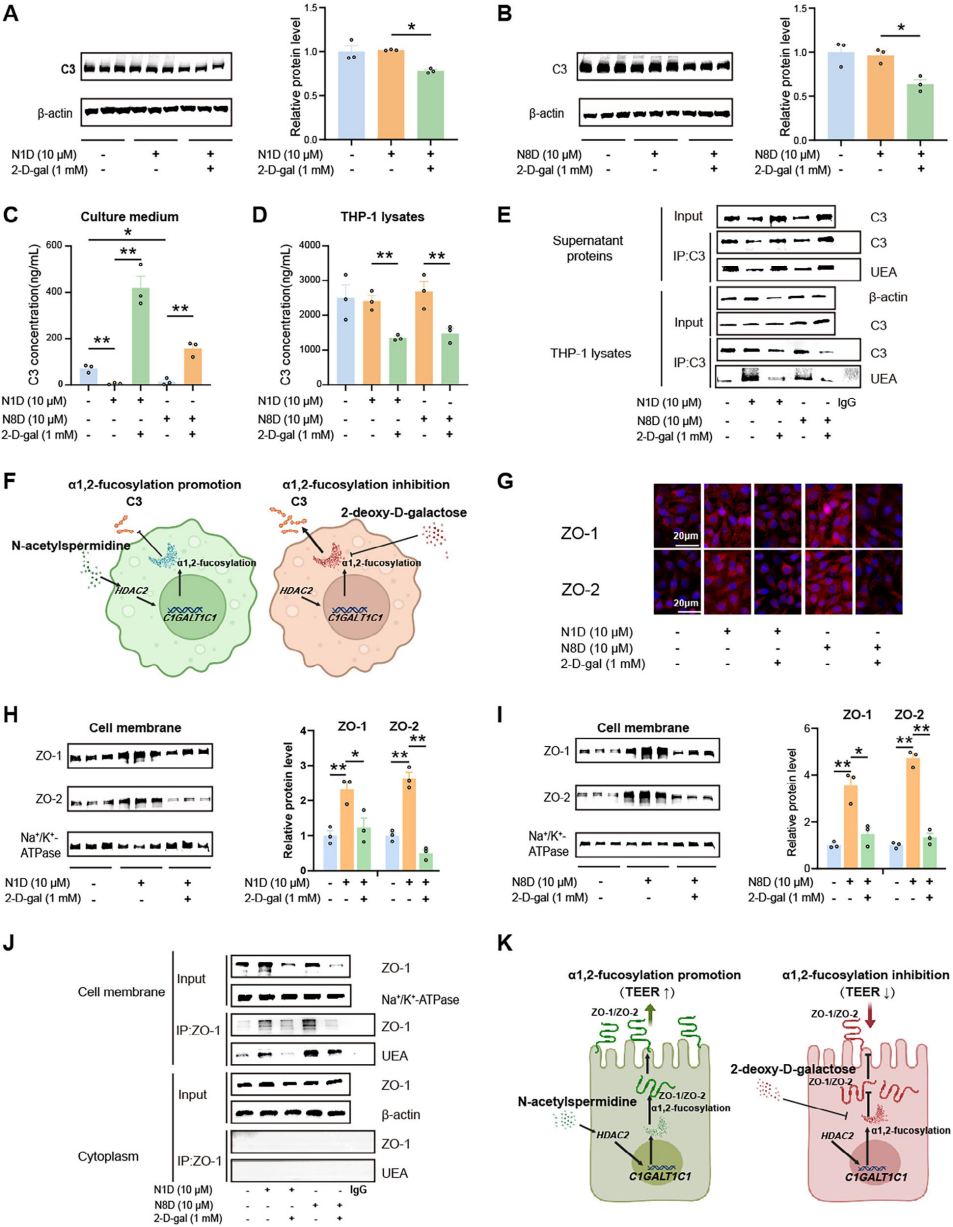
N‐acetylspermidine‐induced α1,2‐fucosylation suppresses C3 secretion and promotes the membrane localization of ZO‐1and ZO‐2. A,B) Immunoblotting analysis of C3 in THP‐1 cells treated with N1D (A) and N8D (B), in combination with 2‐*D‐*gal for 24 h, *n* = 3. C,D) C3 concentrations in the culture media (C) and lysates (D) of THP‐1 cells treated with N1D and N8D, in combination with 2‐*D*‐gal for 24 h, *n* = 3. E) UEA blotting and immunoblotting analysis of cell lysates, supernatant proteins and C3 immunoprecipitant of THP‐1 cells treated with N1D and N8D, in combination with 2‐*D*‐gal for 24 h. F) Mechanism diagram showing that N‐acetylspermidine reduces C3 secretion, which is reversed when α1,2‐fucosylation is inhibited. G) Immunofluorescent staining of ZO‐1 and ZO‐2 in HCoEpic incubated with N1D and N8D for 24 h. H,I) Immunoblotting analysis of ZO‐1 and ZO‐2 in the membrane of HcoEpiC treated with N1D (H) and N8D (I) for 24 h, *n* = 3. J) UEA blotting and immunoblotting analysis of cell membrane and cytoplasmic proteins and ZO‐1 immunoprecipitant of HcoEpiC incubated with N1D and N8D for 24 h. K) Mechanism diagram showing that N‐acetylspermidine facilitates ZO‐1/2 export from the cytoplasm to the membrane, which is impaired upon α1,2‐fucosylation inhibition. Data are representative results of three independent experiments (A–E,G–J). Error bars represent the SEM. Statistical analysis was conducted by one‐way ANOVA followed by Fisher's LSD tests with multiple comparisons adjustments (A–D,H,I). *P < 0.05; **P < 0.01.

Proteomic analysis suggested that α1,2‐fucosylated C3, ZO‐1, and ZO‐2 play essential roles in *A. muciniphila*‐mediated colitis alleviation (Figure 2J–L). Previous studies have shown that posttranslational modifications, such as α1,2‐fucosylation, affect protein localization.^[^ ^6^, ^47^ ^]^ We hypothesized that N‐acetylspermidine may affect C3 secretion by regulating its α1,2‐fucosylation, which is linked to inflammation. Indeed, we found that N‐acetylspermidine significantly reduced C3 concentrations in THP‐1 cell supernatants (**Figure** **7**A–D; Figure S9A,B, Supporting Information). Conversely, inhibiting α1,2‐fucosylation with 2‐deoxy‐*D‐*galactose increased extracellular C3 while decreased intracellular C3 levels (Figure 7C,D). Immunoprecipitation and UEA blotting further confirmed that N‐acetylspermidine increased α1,2‐fucosylation of intracellular C3, while reducing extracellular C3 α1,2‐fucosylation, which was reversed by α1,2‐fucosylation inhibition (Figure 7E). These results indicate that N‐acetylspermidine‐induced α1,2‐fucosylation inhibits C3 secretion (Figure 7F).

ZO‐1 and ZO‐2 are membrane‐associated proteins essential for maintaining intestinal barrier integrity.^[^ ^48^, ^49^, ^50^ ^]^ We proposed that N‐acetylspermidine‐induced α1,2‐fucosylation may promote the precise membrane localization of ZO‐1 and ZO‐2. As expected, N‐acetylspermidine increased the membrane levels of ZO‐1 and ZO‐2 in HcoEpiC, while their cytoplasmic levels remained unaffected (Figure 7G–I; Figure S9C–H, Supporting Information). In contrast, inhibiting α1,2‐fucosylation decreased membrane‐associated ZO‐1 and ZO‐2 levels (Figure 7G–I). Immunoprecipitation and UEA blotting confirmed that N‐acetylspermidine increased α1,2‐fucosylation of membrane‐associated ZO‐1, while reducing it in the cytoplasm, which was reversed by α1,2‐fucosylation inhibition (Figure 7J). The absence of detectable ZO‐1 immunoprecipitation signals corresponds with diminished α1,2‐fucosylation ‐ a biochemical state characteristic of immature protein forms. These findings indicate that N‐acetylspermidine facilitates membrane trafficking of ZO‐1 and ZO‐2 by enhancing their α1,2‐fucosylation (Figure 7K).

## 3 Discussion

**Figure 8:**
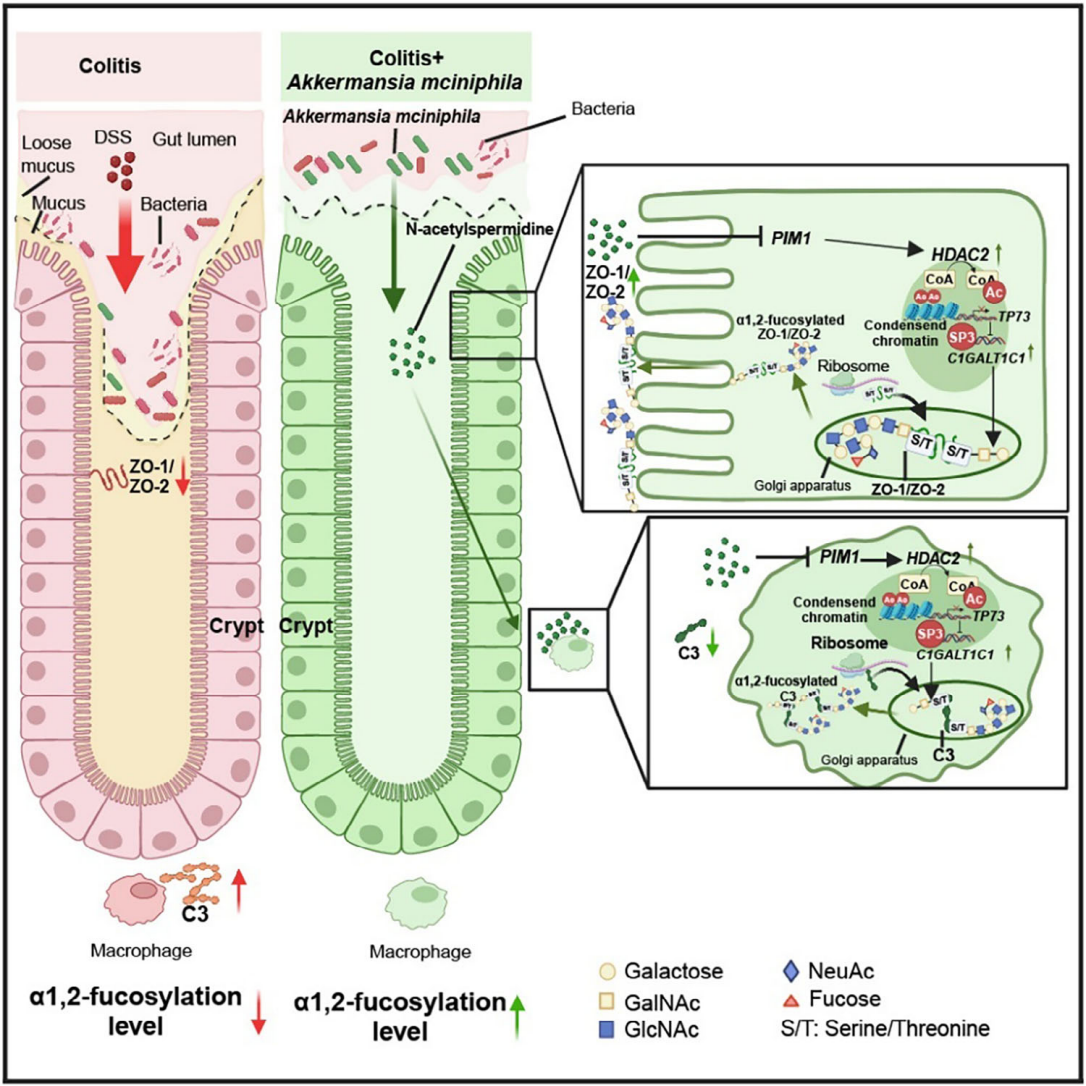
*A. muciniphila* alleviates colitis by enhancing intestinal α1,2‐fucosylation.

The gut microbiota induces intestinal α1,2‐fucosylation, critical for barrier function and immune homeostasis.^[^ ^9^ ^]^ However, the regulatory potential of probiotics and their metabolites on α1,2‐fucosylation remains largely unexplored. In this study, we found that *A. muciniphila* enhances intestinal α1,2‐fucosylation, which is crucial for its colitis‐alleviating effects. Mechanistically, *A. muciniphila*‐derived N‐acetylspermidine inhibits *PIM1*, which increased *HDAC2* expression. This reduces chromatin accessibility and expression of *TP73*, thereby increasing *C1GALT1C1* expression and subsequent α1,2‐fucosylation. Furthermore, elevated α1,2‐fucosylation promotes membrane localization of ZO‐1 and ZO‐2, while inhibiting C3 secretion, contributing to colitis alleviation. Together, our findings provide insights into the mechanisms by which probiotics alleviate colitis by modulating intestinal glycosylation (**Figure** **8**).

ILC3s‐derived IL‐22, induced by gut microbiota, is a key signal in promoting intestinal α1,2‐fucosylation by upregulating *FUT2* expression.^[^ ^9^ ^]^ However, *A. muciniphila* CCFM1079 decreased colonic IL‐22 expression, without affecting *Fut2* expression, while significantly enhancing intestinal α1,2‐fucosylation. This suggests that the α1,2‐fucosylation mediated by *A. muciniphila* was not solely dependent on the IL‐22‐*Fut2* pathway. Similarly, in wild‐type mice, α1,2‐fucosylation was elevated in the cecum compared to *Il22ra1* ^‐/‐^ mice, with no changes in IL‐22 or *Fut2* expression.^[^ ^51^ ^]^ We found that *A. muciniphila* CCFM1079 significantly upregulated *C1galt1c1* expression alongside α1,2‐fucosylation levels, highlighting the role of *C1galt1c1* in regulating α1,2‐fucosylation.

*A. muciniphila* increased α1,2‐fucosylation, without affecting α1,6‐fucosylation levels. The distinct regulation may be due to the fact that *C1galt1c1* is responsible for synthesizing core 1 structure on O‐glycans, providing sites for α1,2‐fucosylation, without effects on the α1,6‐fucosylation that only occurs on N‐glycans.^[^ ^52^ ^]^ *C1galt1* ^–/–^ mice, which lack fucosylated core 1 structures, develop spontaneous colitis, emphasizing the importance of terminal α1,2‐fucosylation on core 1 in colitis prevention.^[^ ^53^ ^]^ Additionally, *C1GALT1C1* has been identified as a candidate risk gene associated with both CD and UC, suggesting its key role in IBD pathogenesis.^[^ ^1^ ^]^

We found that *A. muciniphila*‐derived N‐acetylspermidine upregulated *C1GALT1C1* expression. Similarly, Grajeda‐Iglesias et al. observed that administration of *A. muciniphila* increased intestinal N‐acetylspermidine concentrations.^[^ ^54^ ^]^ Furthermore, N‐acetylspermidine enhanced α1,2‐fucosylation and barrier function in two human cell lines and *C. elegans*, suggesting a conserved effect across species. Mechanistically, N‐acetylspermidine upregulates *HDAC2* via *PIM1* inhibition, which reduces chromatin accessibility at the *TP73* locus, thereby promoting *C1GALT1C1* expression and consequent α1,2‐fucosylation. Emerging evidence demonstrates the protective role of PIM1 inhibition in colitis alleviation. These findings position PIM1 inhibitors (e.g., N‐acetylspermidine) as promising therapeutic candidates for colitis intervention. Additionally, *A. muciniphila* ameliorates colorectal tumourigenesis through its secreted protein Amuc_2172, which can enter cancer cells and acetylate histone H3 at Lys14.^[^ ^11^ ^]^ Amuc_2172 is a GCN5‐related N‐acetyltransferase that converts spermidine to N‐acetylspermidine.^[^ ^55^ ^]^ Therefore, *A. muciniphila*‐mediated histone deacetylation and acetylation may contribute to a dynamic balance in histone acetylation levels, highlighting the complex and nuanced roles of its acetylation pathways in host epigenetic regulation, warranting further investigation.

Our study found that *A. muciniphila* upregulated intestinal ZO‐1/ZO‐2 and reduced C3 in colitis mice. Similarly, derivatives of *A. muciniphila* (propionic acid, Amuc_1100), upregulate ZO‐1 expression.^[^ ^56^, ^57^, ^58^ ^]^ However, in vitro experiments demonstrated that *A. muciniphila*‐derived N‐acetylspermidine promoted membrane localization of ZO‐1/ZO‐2, and suppressed C3 secretion by enhancing α1,2‐fucosylation, without altering their expression. This apparent discrepancy suggests that *A. muciniphila* regulates ZO‐1, ZO‐2, and C3 via synergistic interactions among its bioactive components, necessitating further studies to elucidate multifactorial mechanisms underlying its colitis‐alleviating networks.

Emerging glycomics technology is pivotal in understanding the roles of glycoproteins in IBD.^[^ ^1^, ^59^ ^]^ For instance, Yao et al. employed N‐glycoproteomics to reveal the role of sialylation in protecting MUC2 from bacterial proteolytic degradation, which maintains gut integrity in colitis.^[^ ^60^ ^]^ Despite these advances, functional α1,2‐fucosylated proteins in colitis remain understudied. Hence, we identified altered α1,2‐fucosylated proteins in the colons of colitis mice and inflamed tissues of CD patients, which are involved in complement and coagulation cascades and tight junctions. We further observed that decreased α1,2‐fucosylation facilitated C3 secretion. A previous study found that charged residues such as sialic acid and sulfate on O‐glycans can interact with Ca^2+^, facilitating the secretion of secretory glycoprotein MUC2.^[^ ^61^ ^]^ Similarly, C3 secretion is dependent on intracellular Ca^2+^ concentration.^[^ ^62^ ^]^ Therefore, we speculated that decreased α1,2‐fucosylation of C3 may accompany increased sialic acid and sulfate that can interact with Ca^2+^, thus potentially promoting its secretion. In contrast, increased α1,2‐fucosylation facilitated the location of membrane‐associated proteins ZO‐1 and ZO‐2 to the cell membrane. Similarly, a previous study showed that FUT8‐mediated fucosylation promotes the export of the membrane‐bound protein MUC1 to the apical plasma membrane.^[^ ^6^ ^]^ These findings suggest differential roles of α1,2‐fucosylation in the trafficking of secretory and membrane‐associated proteins.

In addition to N‐acetylspermidine, *A. muciniphila* also produces D‐serine, which was elevated in the cecum of colitis mice treated with *A. muciniphila*. Our recent study demonstrated that D‐serine mitigates colitis via the upregulation of intestinal α1,2‐fucosylation, which helps improve gut microbiota imbalance and maintains gut barrier function.^[^ ^63^ ^]^ However, correlation analysis showed that cecal *A. muciniphila* abundance was not associated with D‐serine concentration, suggesting that other commensal bacteria may contribute to D‐serine production. These findings underscore the role of gut microbiota‐derived metabolites in regulating host post‐translational modifications.

In conclusion, this study provides insights into the mechanisms of how gut commensals ameliorate colitis by regulating host glycosylation, underscoring the application potential of *A. muciniphila* and N‐acetylspermidine in developing strategies for colitis.

## 4 Experimental Section

### Colon Tissues of Patients with Crohn's Disease

Paired normal and inflamed colon tissues from four Crohn's disease (CD) patients were surgically excised at the Affiliated Hospital of Jiangnan University and immediately preserved in liquid nitrogen. Informed consent was obtained from all patients, and the study was approved by the Jiangnan University Medical Ethics Committee (JUN202406RB023).

### Strains of *A. muciniphila*

*A. muciniphila* ATCC BAA835, CCFM1079 (FSDLZ36M5), CCFM1081 (FSDLZ39M14), CCFM1082 (FSDLZ20M4) were cultured in brain heart infusion broth (Qingdao Hope Bio‐Tcehnology Company, China) containing 0.25% type II mucin from porcine stomach (Sigma) under strict anaerobic conditions at 37 °C.^[^ ^15^ ^]^ The bacteria were collected and preserved at −80 °C with 20% glycerol until further use.

### Induction of Experimental Colitis in Mice

Male C57BL/6J mice (6 to 8 weeks old) were bought from Beijing Vital River Laboratory. All mice were housed in a controlled environment with 12‐hour light/dark cycles, a temperature range of 22–24 °C, humidity between 40–70%, and provided with enriched water and ad libitum food under specific‐pathogen‐free conditions.

Two sets of interventions were performed:

1) Colitis mice gavaged with *A. muciniphila*: From week 1 to week 2, all mice received drinking water supplemented with an antibiotic cocktail (1 g L^−1^ of ampicillin, neomycin, metronidazole, and 0.5 g L^−1^ of vancomycin) (Figure 1G). Acute colitis was induced by adding 3% dextran sodium sulfate to the drinking water during week 6. For the *A. muciniphila* intervention, colitis mice were gavaged with 200 µL PBS containing 1 × 10^9^ CFU *A. muciniphila* strains ATCC BAA‐835, CCFM1079, CCFM1081, and CCFM1082 every two days from week 2 to week 6.

2) α1,2‐fucosylation inhibition in colitis mice gavaged with *A. muciniphila* CCFM1079: Acute colitis was induced by adding 3% DSS in the drinking water daily during the final week (Figure 1P). Two groups of mice were gavaged with 200 µL PBS containing 1 × 10^9 ^CFU *A. muciniphila* CCFM1079, with one group being intraperitoneally injected with 2‐deoxy*‐D‐*galactose every other day for 4 weeks (from week 1 to week 4). All experimental protocols received approvals from the Ethics Committee of Jiangnan University, China (JN. No. 20220615c1001225 and JN. No. 20230915c0961225).

### Colitis of Caenorhabditis Elegans and Supplementation of *N*‐Acetylspermidine

The model animal *Caenorhabditis elegans* (*C. elegans*) wild‐type strain N2 was purchased from SHANGHAI MODEL ORGANISMS and maintained on nematode growth media (NGM) at 20 °C, using *Escherichia coli* OP50 (grown in LB Broth Lennox at 37 °C) as the food source. To investigate the effect of N‐acetylspermidine on the intestinal barrier function of *Caenorhabditis elegans* (*C. elegans*), the worms were treated with 10 µm N1‐acetylspermidine and N8‐acetylspermidine for 48 h. Subsequently, colitis was induced by treatment of 50 µg mL^−1^ lipopolysaccharide (LPS) for 24 h.^[^ ^64^, ^65^ ^]^ Additionally, N‐acetylspermidine refered to both N8‐ and N1‐acetylspermidine when the site of acetylation was not specified.

### The Assay of IL‐22 and C3 Using ELISA

The Mouse/Rat lL‐22 Quantikine ELISA Kit (R&D systems, USA) was used to measure IL‐22 levels in the colon tissues of colitis mice. The concentration of C3 in the supernatants of THP‐1 cells was detected using the Human C3a ELISA Kit (PC091, Beyotime, China).

### H&E Staining of Colon Tissues

The colons of mice were fixed in 10% formalin. After paraffin embedding, the colon tissues were sectioned at a thickness of 4 µm for hematoxylin and eosin (H&E) staining to examine the pathology. Images were captured using the PANNORMIC MIDI (3D HISTECH, Hungary). Histological evaluation was performed in a blinded manner according to the established standards based on severity, extent, damage, inflammation, and regeneration.^[^ ^66^ ^]^

### Cecal Microbiome Analysis Based on 16S rRNA Gene Sequencing

After mouse dissection, the cecal contents were carefully collected, transferred into sterile cryovials, rapidly quenched in liquid nitrogen, and subsequently stored at –80 °C The DNA of cecal contents was extracted using A FastDNA Spin kit (MP Biomedicals Ltd., USA), and the amplification of V3‐V4 regions of the bacterial 16S rRNA were conducted using 2×Taq MasterMix (CWBIO, China) with primers 341F and 806R with a barcode (Table S8, Supporting Information). Then, the purification of PCR products was performed using a DNA Gel/PCR Purification Miniprep Kit (Beiwo Meditech Co., Ltd., China). Libraries were constructed using the Library Prep Kit for Illumina (Illumina, United States) and sequenced by Illumina NextSeq 2000. Automated cluster generation and 2 × 250‐bp paired‐end sequencing with dual‐index reads were performed, producing demultiplexed fastq‐files. Bioinformatics analysis of the 16S rRNA gene amplicons was conducted using QIIME 2 (version 2018.6.0).^[^ ^67^ ^]^ Briefly, fastq reads were processed using the dada2 program, using the denoise‐paired command to remove low‐quality reads. Dada2 generated unique features to enable comparisons across studies. Taxonomic assignment of these features was conducted with the GreenGenes reference database (version 13‐2) classifier, applying a similarity threshold of 99%. Furthermore, calculations of alpha and beta diversities were performed within the QIIME2 environment. The linear discriminant analysis (LDA) Effect Size (LEfSe) was next employed to identify taxa exhibiting the highest abundance in particular groups compared to other groups.^[^ ^68^ ^]^ To determine the association between gut microbiota and other indexes including transcription levels of genes in colon tissues and the relative concentration of metabolites in cecal contents of mice, a correlation analysis based on the Spearman correlation was constructed in R (version 3.6.3). To Determine the abundance of *A. muciniphila* in the cecum, its level was measured using QuantiNova SYBR Green PCR master mix (QIAGEN, German) on a CFX96 Touch Real‐Time PCR Detection System (Bio‐Rad, USA) with specific primers for *A. muciniphila*. The primers for universal bacteria served as controls. The sequences of primers are shown in Table S8 (Supporting Information).

### Untargeted Metabolomics Analysis of Cecal Contents of Mice

For untargeted metabolomics analysis, the sample preprocessing of cecal contents including protein precipitation was conducted as previously described.^[^ ^63^ ^]^ A Quality Control (QC) sample was prepared by pooling equal volumes from all samples, and a blank sample was prepared by mixing acetonitrile and water (1:1, v/v). The samples were then analyzed using ultra‐performance liquid chromatography using a Q‐Exactive high‐resolution mass spectrometer (Thermo Fisher Scientific, USA), equipped with a C18 column (2.6 µm, 2.1 × 100 mm, Phenomenex, UHPLC Kinetex). The mobile phase consisted of (A) 0.01% acetic acid in water and (B) 50% acetonitrile in isopropanol. The injection volume was 2 µL, and the flow rate was set to 300 µL min^−1^. Full MS scan mode was used in both positive and negative ionization modes for each sample. Peak identification, peak extraction, and peak alignment of the metabolic data were conducted using Compound Discoverer 3.2 (Thermo Fisher Scientific Inc., USA). Calculation of the *P* value and log_2_ fold change was conducted using MetaboAnalyst 5.0. Metabolites meeting the following criteria were identified as differential metabolites: *P* < 0.05, |log_2_ fold change| > 1.

### Lectin Blotting and Immunoblotting

Proteins of cells were extracted using a mammalian protein extraction reagent (CWBio, China), and RIPA Lysis Buffer (CWBio, China) was utilized for the extraction of total proteins from animal tissues. The protein concentration was determined using the BCA protein assay kit (Beyotime, China). Proteins were resolved by SDS‐PAGE and transferred onto PVDF membranes (Millipore, USA). The membranes were blocked for 1.5 h in TBST (Tris‐buffered saline with 0.1% Tween‐20) containing 5% (w/v) bovine serum albumin. To detect α1,2‐fucosylation and α1,6‐fucosylaion levels, the membranes were incubated at room temperature for 1.5 h with 2 µg mL^−1^ biotinylated Ulex Europaeus Agglutinin I (UEA I, Vector laboratories, USA) or biotinylated Aleuria Aurantia Lectin (AAL, Vector laboratories, USA). The membranes were then incubated with an HRP‐linked antibody (anti‐biotin) (Cell Signaling Technology, USA) for 1.5 h at room temperature. For detecting the level of proteins using immunoblotting, the membranes were incubated overnight at 4 °C with primary antibodies followed by incubation with goat anti‐rabbit/mouse secondary antibodies (Proteintech). Protein bands were detected using the ECL method (Millipore, USA), and band intensities were measured with Image J2.^[^ ^69^ ^]^

### Identification of α1,2‐Fucosylated Proteins

For the identification of α1,2‐fucosylated proteins in the colons of mice and CD patients, UEA‐1 precipitation and mass spectrometry were performed as previously described.^[^ ^70^ ^]^ Briefly, the proteins of the colons were extracted using RIPA Lysis Buffer (CWBio, China). Agarose‐bound Ulex Europaeus Agglutinin I (UEA I), (AL‐1063‐2, Vector laboratories, USA) was drawn into the Bio‐Spin Chromatography Columns (732‐6008, Bio‐Rad, USA) and washed with 10 column volumes of HBS buffer (10 mm HEPES, 0.15 m NaCl, pH 7.5). The proteins were incubated with agarose‐bound UEA I in the sealed columns for 45 min. Non‐binding proteins were removed by washing with HBS buffer 2–3 times, and then the UEA‐binding proteins (α1,2‐fucosylated proteins) were released from the agaroses by washing with 200 mm L‐fucose (S‐9007, Vector laboratories, USA). α1,2‐fucosylated proteins were collected and reduced with 5 mM dithiothreitol for 30 min at 56 °C, then alkylated with 11 mm iodoacetamide for 15 min at room temperature in the dark. The proteins were subsequently diluted with 200 mm TEAB to reduce the urea concentration to below 2 m. Trypsin was added at a 1:50 trypsin‐to‐protein mass ratio for overnight digestion, followed by a second digestion at 1:100 ratio for 4 h. Peptides were desalted using a Strata X SPE column, and the resulting tryptic peptides were prepared for further LC‐MS/MS analysis (PTMBio company, China). Gene Ontology analysis and The Kyoto Encyclopedia of Genes and Genomes annotation were conducted to identify the α1,2‐fucosylated proteins. Proteins exhibiting a *CV* (Standard coefficient of variation) <0.1 were deemed differentially expressed.

### Measurement of Intestinal Integrity of Caenorhabditis Elegans by Smurf Assay

The worms were stained with brilliant blue (5%, w/v) for 2 h and then washed with M9 buffer until the blue dye was no longer visible. The worms were mounted on a thin layer of 2% (wt/vol) agarose containing 0.25 m levamisole hydrochloride to anesthetize them. Afterward, the worms were observed under an Axio Imager Z2 microscope (ZEISS, Germany). The leakage rate of brilliant blue in the body cavity was analyzed using Image J2.^[^ ^69^ ^]^

### Lectin Staining of Caenorhabditis Elegans

The worms were incubated in PBS containing 0.2% bovine serum albumin and 10 µg mL^−1^ rhodamine‐conjugated Ulex Europaeus Agglutinin I (UEA I) for 2 h at 20 °C. Then, the worms were washed three times with PBS to remove unbound UEA and mounted on a thin layer of 2% (wt/vol) agarose containing 0.25 m levamisole hydrochloride for anesthesia. The worms were subsequently observed under an Eclipse 80i microscope (Nikon, Japan). The fluorescence intensity was analyzed using ImageJ.

### Lectin Blotting and Silver Staining of Caenorhabditis Elegans

The total proteins of *C. elegans* were extracted using RIPA Lysis Buffer (CWBio, China). The protein concentration was measured using the BCA protein assay kit (Beyotime, China) and the concentrations of all samples were adjusted to be consistent. A Protein Silver Stain Kit (CWBio, China) was used to verify that each sample contained a consistent amount of protein. Lectin blotting was performed as described in the “Immunoblotting and Lectin Blotting in Mice” section.

### THP‐1, RAW264.7 and HcoEpiC Cell Lines

The human leukemia monocyte cell line (THP‐1) and mouse monocytic macrophagic cell line (RAW264.7) were grown in RPMI 1640 medium (Gibco). Human colonic epithelial cells (HcoEpiC) were cultured in complete DMEM medium. 10% fetal bovine serum and 1% penicillin/streptomycin were added to the cell culture media and cells were incubated at 37 °C in a 5% CO_2_ atmosphere. THP‐1 cells were induced to an adherent macrophage‐like phenotype by incubating with 200 ng mL^−1^ Phorbol 12‐myristate 13‐acetate (PMA, Sigma) for 48 h.

### Co‐Culture of HcoEpiC with *A. muciniphila*

Before treatment with *A. muciniphila*, the cell culture media were replaced with antibiotic‐free media. HcoEpiC were then co‐cultured with *A. muciniphila* at a multiplicity of infection (MOI) of 100 for 4 h.

### Treatment of RAW264.7 Cells with Serum from Colitis Mice

The RAW264.7 cells were incubated with serum from colitis mice and those treated with *A. muciniphil* at a concentration of 2.5% for 24 h. The cells were harvested for RNA extraction, followed by RNA‐sequencing.

### α1,2‐Fucosylation Inhibition and PIM1 Inhibition of HcoEpiC and THP‐1 Cells

To assess the inhibitory effects of 2‐deoxy‐*D*‐galactose on α1,2‐fucosylation, HcoEpiC and THP‐1 cells were incubated with 0, 0.1, 1, and 10 mm of 2‐deoxy‐*D*‐galactose for 24 h. Based on the results, 1 mm 2‐deoxy‐*D*‐galactose, showing the most significant inhibitory effect, was used to inhibit α1,2‐fucosylation in HcoEpiC and THP‐1 cells (Figure S8A,B, Supporting Information). PIM1 inhibition of HcoEpiC and THP‐1 cells was induced using 1 µm AZD1208 and 10 µm SGI1776 for 12 h.^[^ ^71^, ^72^ ^]^

### Treatment of N‐Acetylspermidine in HcoEpiC and THP‐1 Cells

To investigate the effects of N‐acetylspermidine on α1,2‐fucosylation, HcoEpiC and THP‐1 cells were treated with 0, 0.1, 1, and 10 µm of N1‐acetylspermidine and N8‐acetylspermidine for 24 h. Consequently, 10 µm showing the most significant promoting effect on α1,2‐fucosylation, was chosen for further experiments (Figure 4D–G).

### Transepithelial Electronic Resistance Assay

HcoEpiC were seeded in 12‐well Transwell plates (0.4 µm pore size, PET membrane, Corning, USA) at a density of 5 × 10^5^ cells mL^−1^ and cultured until reaching tight confluence. The Millicell ERS‐2 device (Merk, USA) was used to measure the TEER following 48 h of N1D and N8D treatment. Readings were obtained from three separate wells for each experimental condition.

### Knockdown of HDAC1, HDAC2, and PIM1 in THP‐1 Cells and HcoEpiC

Expression of *HDAC1*, *HDAC2*, and *PIM1* in THP‐1 cells and HcoEpiC was interfered with siRNAs specific for *HDAC1*, *HDAC2*, and *PIM1*, which were bought from the GenePharma company (China). The siRNA sequences are listed in Table S7 (Supporting Information). The transfection of HcoEpiC was carried out using GP‐transfect‐Mate (G04008, GenePharma, China), which contained 20 µm siRNA. Transfection was performed on cell suspensions at 60–80% confluence. After six hours of transfection, the cells were replenished with fresh medium and used for further experiments. For the transfection of THP‐1 cells, CALNP RNAi in vitro (DN001, D‐Nano Therapeutics, China) containing 20 µm siRNA was added into cell suspensions at a cell density of 5 × 10^5^ cells mL^−1^ and incubated for 24 h. After transfection, the cells were used for further experiments.

### Assays for Transposase‐Accessible Chromatin Using Sequencing (ATAC‐Seq)

1–5 × 10^4^ THP‐1 cells were treated with cell lysis buffer, and the nuclei were collected by centrifugation at 500 g for 5 min. Transposition and library preparation for high‐throughput DNA sequencing were conducted using the TruePrep DNA Library Prep Kit V2 for Illumina kit (Catalog NO. TD501, Vazyme). The library products were enriched, quantified, and sequenced on the Novaseq 6000 platform (Illumina) using the PE150 model at SeqHealth (Wuhan, China). Raw sequencing data were filtered using fastp (version 0.23.1), with low‐quality reads removed and adaptor‐contaminated sequences trimmed.^[^ ^73^ ^]^ Clean reads were then mapped to the human reference genome (GRCh38_release 110) using bowtie2 (version 2.2.6) with default parameters.^[^ ^74^ ^]^ Sambamba (version 0.7.1) was used for SAM/BAM format conversion and removal of PCR duplicate reads.^[^ ^74^ ^]^ Read distribution analysis was performed using RSeQC (version 2.6).^[^ ^75^ ^]^ Insert length was calculated with the Collect Insert Size Metrics tools from picard software (version 2.8.2). DeepTools (version 2.4.1) was used to visualize the distribution of reads on upstream and downstream of the TSS.^[^ ^76^ ^]^ Peak calling was performed using MACS2 (version 2.1.1),^[^ ^77^ ^]^ while Bedtools (version 2.30.0) was employed for peak annotation and distribution analysis. Differential peaks were identified using csaw (version 1.24.3).^[^ ^78^ ^]^ Gene ontology (GO) and Kyoto Encyclopedia of Genes and Genomes (KEGG) analyses were conducted on the annotated genes using KOBAS (version 2.1.1), with a corrected *P‐*value threshold of 0.05 to identify statistically significant enrichments.^[^ ^79^ ^]^

### Extraction of Membrane, Cytoplasmic, and Secretory Proteins

Membrane and cytoplasmic proteins from HCoEpic were extracted using the Mem‐PER Plus Membrane Protein Extraction Kit (Thermo Scientific, USA). Secretory proteins from THP‐1 cells were collected using an Amicon Ultra Centrifugal Filter, 30 kDa MWCO (Millipore, USA).

### Immunoprecipitation

Equal amounts of protein (200–500 µg per sample) were pre‐cleared with protein A and protein G agarose beads (absin, abs955) at 4 °C for 45 min. Immunoprecipitation of ZO‐1 and C3 was performed using ZO‐1 antibody (abcam, ab276131) and C3 antibody (abcam, ab97462) overnight at 4 °C. A Rabbit IgG Isotype Control (absin, abs172294) was used as a negative control. Protein A and protein G agarose beads were added to the mixture and incubated at 4 °C for 3 h. After centrifugation at 10 000 g for 1 min, the beads were washed three times and then boiled in SDS sample buffer at 95 °C for 5 min. The supernatants were used for immunoblotting detection using ZO‐1, C3, and anti‐UEA antibodies (Vector laboratories, AL‐1063‐2).

### Metagenomic Data Analysis

Metagenomic data from stool samples of 103 IBD patients and 41 non‐IBD individuals were obtained from the National Center for Biotechnology Information (NCBI) with accession number PRJNA385949.^[^ ^23^ ^]^ Human reads were filtered using KneadData with default parameters (https://github.com/biobakery/kneaddata). Subsequently, bacterial abundance was quantified using MetaPhlAn (version 4.1.0).^[^ ^80^ ^]^ Gene abundance was measured with HUMAnN (version 3.9) utilizing the UniRef90 database with default settings.^[^ ^81^ ^]^ The Log_2_ (gene expression) was used to assess changes in genes related to N‐acetyltransferase between IBD patients and non‐IBD individuals. To avoid taking the logarithm of zero, the values of gene expression were multiplied by 1000, and 1 was added before calculating the log fold change.

### Single‐Cell Transcriptome Data Analysis

The analysis of single‐cell transcriptome data was conducted as described previously.^[^ ^30^ ^]^ Briefly, single‐cell FASTQ files were mapped to the human genome (GRCh38‐3.1.0) using Cell Ranger (v64.0.0), yielding gene‐by‐cell count matrices. Quality control and analysis, including normalization, integration, and clustering, were performed in Seurat (v3.2). Cells with fewer than 1000 detected genes, over 10% mitochondrial reads, or more than 50 000 total reads were excluded. For clustering, the authors focused on the top 2000 variable genes and principal components, with immune clusters removed to analyze epithelial cells. Highly enriched markers identified clusters, and cell types were annotated based on known markers. Differential expression analysis between IBD and non‐IBD samples used the Wilcoxon rank‐sum test.

### Untargeted Metabolomics Analysis of the Supernatants from *A. muciniphila*

The culture media of *A. muciniphila* ATCC BAA‐835, CCFM1079, CCFM1081, and CCFM1082 were centrifuged at 10 000 × g at 4 °C for 5 min, and then 100 µL supernatant was collected for untargeted metabolomics analysis. For the precipitation of proteins, 400 µL of a pre‐cooled methanol and acetonitrile (1:1 volume) mixture at −20 °C was added, followed by vortexing for 30 s. The mixture was then sonicated at 4 °C for 10 min. Samples were subsequently incubated at −20 °C for 1 h and centrifuged at 15 000 rpm for 15 min at 4 °C. The remaining steps followed the procedures detailed in the section titled “Untargeted Metabolomics Analysis of Cecal Contents in Mice.”

### Complete Genome Sequencing of A. muciniphila

Universal primers 27F and 1492R were used for species identification by targeting the 16S rRNA gene (Table S8, Supporting Information). For complete genome sequencing of *A. muciniphila* CCFM1079, strain CCFM1081, and strain CCFM1082, genomic DNA was extracted using the Fast DNA Spin Kit for Feces (MP Biomedicals, USA). Subsequently, the TELL‐Seq WGS Library Prep kit (Universal Sequencing Technology Corp, China) was employed to process the genomic DNA, generating complete DNA libraries. These libraries were then sequenced on the Novaseq 6000 platform at Universal Sequencing Technology Corp (China). The quality of the raw sequencing data was assessed with FastQC (v0.11.9). Cutadapt (v1.0.3) was utilized to trim and eliminate potential sequencing adapters from the raw reads. Additionally, reads containing low‐quality bases or a high proportion of ambiguous bases were filtered out, along with erroneous barcodes and their corresponding sequences. Clean reads were assembled using Tell‐Link to generate scaffold sequences.^[^ ^82^ ^]^ Prokka was employed for gene prediction on the scaffold sequences, providing information on both protein‐coding and non‐coding genes.^[^ ^83^ ^]^ Functional annotation of the protein‐coding genes was carried out using NR, GO, COG, KEGG, and Swiss‐Prot databases.^[^ ^84^, ^85^, ^86^ ^]^

### Pangenome Analysis

The protein sequences of complete genomes of *A. muciniphila* strains were input into the Bacterial Pan Genome Analysis tool (BPGA) v1.3 for pangenome analysis.^[^ ^87^ ^]^ Genes were then clustered into orthologous groups using the ultra‐fast USEARCH clustering algorithm (v11.0.667) with a sequence similarity threshold of 50%. Phylogenetic trees were constructed based on the pan‐genome and core‐genome using the neighbor joining (NJ) method in BPGA. Gene function annotation was performed using the KEGG database.^[^ ^88^ ^]^

### RNA‐Seq Analysis

Total RNA was extracted using the RNAprep Pure Cell Kit (TIANGEN, Beijing, China). Subsequently, library preparations were then sequenced on the Illumina Novaseq 6000 platform, and the raw sequencing data quality was assessed with FastQC. RNA STAR (version 2.7.0e) was used to map the fastq files to the mm10 and hg38 reference genome.^[^ ^89^ ^]^ Gene expression pseudo‐counts were generated from the resulting BAM files with FeatureCounts.^[^ ^90^ ^]^ Calculation of transcripts per kilobase million values for each gene was conducted using edgeR (version 3.7).^[^ ^91^ ^]^ Genes exhibiting a log _2_ (fold change) ≥ 1 or log _2_ (fold change) ≤ −1, in conjunction with *P*‐values < 0.05, were considered differentially expressed. The RNA‐seq data generated in this study were deposited in the Big Submission Portal of the National Genomics Data Center (https://ngdc.cncb.ac.cn/gsub/) and the accession numbers were PRJCA026182 and PRJCA026197.

### Real‐Time PCR

Total RNA was extracted from cells using the RNAprep Pure Cell Kit (TIANGEN, Beijing, China). cDNA was generated using HiScript III All‐in‐one RT SuperMix (R333, Vazyme, China). Quantitative PCR (qPCR) was conducted using the ChamQ Universal SYBR qPCR Master Mix (Q711, Vazyme, China) on the CFX96 Touch Real‐Time PCR Detection System (Bio‐Rad, USA). The primer sequences utilized for RT‐qPCR analysis can be found in Table S8 (Supporting Information). Relative expression to *ACTB* was calculated.

### Prediction of the Transcriptional Regulatory Factors

To predict the transcriptional regulatory factors of *C1GALT1C1*, its promoter region sequence was downloaded from NCBI and input to the Transcription Factor Prediction tool of AnimalTFDB (https://guolab.wchscu.cn/AnimalTFDB#!/predict).

### Spearman Correlation Analysis Based on GEPIA2 Database

Spearman correlation analysis of *PIM1*, *HDAC2*, and *C1GALT1C1* in the small intestine, sigmoid colon, transverse colon, and control of colon adenocarcinoma was performed using the GEPIA2 web server, based on TCGA and GTEx databases.^[^ ^39^ ^]^

### Molecular Docking

The protein crystal structure of PIM1 was obtained from the Protein Data Bank (PDB) database, and the structure of N8‐acetylspremidine was downloaded from the PubChem database of NCBI. Molecular docking of PIM1 with N8‐acetylspremidine was performed using the software AutoDock (version 4.2).^[^ ^92^ ^]^ Prior to docking, water molecules were removed, and hydrogenation, steric hindrance optimization, and hydrogen bond optimization were applied to PIM1 to resolve any stereoscopic conflicts. PIM1 was designated as the receptor, and the N8‐acetylspremidine as the ligand. Docking parameters related to the protein's surface structure and surface potential were set accordingly, while all other parameters were kept at default values, except for the number of runs, which was increased to 100 for each test. The docking results were grouped into clusters based on root‐mean‐square deviation (RMSD) criteria, and the conformations within each cluster were ranked by energy levels.

### Statistical Analysis

Unless otherwise specified, statistical analyses were performed using GraphPad Prism (version 9.2.0). The Shapiro–Wilk test was initially used to assess the normality of the data. For normally distributed data (presented as means ± SEM), comparisons between two groups were made using unpaired Student's *t*‐tests, and comparisons among three or more groups were conducted using one‐way analysis of variance (ANOVA) followed by Fisher's LSD tests with adjustments for multiple comparisons. For non‐normally distributed data (presented as Median (IQR)), comparisons between two groups were performed using the Mann–Whitney *U*‐test, and comparisons among three or more groups were made using the Kruskal–Wallis test followed by uncorrected Dunn's test with adjustments for multiple comparisons. Categorical data, such as H&E pathohistological scores, are presented as counts with percentages and compared between groups using chi‐square tests. The coefficient of variation (*CV*) was used to statistically analyze the expression levels of α1,2‐fucosylated proteins in colitis mice detected by proteomics. Statistical significance of RNA‐seq data was determined by edgeR. PCA was performed and plotted in R (version 3.6.3). Differentially expressed genes were displayed in heatmaps, with data standardized using Z‐scores. Correlation analysis was conducted using the Spearman method. Statistical significance was set at *P *< 0.05.

## Conflict of Interest

The authors declare no conflict of interest.

## Author Contributions

Y.Y. and Z.P. contributed equally to this work. Y.Y., Z.P., J.Z., Q.Z., W.C., and W.L. designed the research. Y.Y., Y.D., and Z.C. conducted the experiments. Y.C. and Y.Y. performed formal analysis and methodology development. Z.P. and Z.C. contributed to software implementation. J.Z., Q.Z., and W.C. provided resources and validation. H.W., Q.Z., and W.C. acquired funding. Y.Y. prepared visualizations and wrote the original draft. W.L. supervised the project and revised the manuscript. All authors reviewed and approved the final manuscript.

## Supporting information



Supporting Information

Supplemental Table 1

Supplemental Table 2

Supplemental Table 3

Supplemental Table 4

Supplemental Table 5

Supplemental Table 6‐8

Supplemental Table 9

## Data Availability

All raw data for 16S rRNA gene sequencing, RNA‐seq, ATAC‐seq, and complete genome sequencing have been deposited in the Genome Sequence Archive (GSA) of the National Genomics Data Center (NGDC), China National Center for Bioinformation (CNCB). The accession numbers for all datasets are available in the Table S9.
