# Supplementary material for: Akkermansia muciniphila‐Derived N‐Acetylspermidine Modulates the Localization of Intestinal α1,2‐Fucosylated Proteins to Maintain Gut Homeostasis
Source: Adv Sci (Weinh). 2025 Aug 7;12(38):e06576. doi: 10.1002/advs.202506576 (PMC12520552; doi:10.1002/advs.202506576)
Supplement: Supplementary file 1 — Supporting Information [file ADVS-12-e06576-s005.docx]

**Supplemental information**

**Akkermansia muciniphila-Derived N-acetylspermidine Modulates the Localization of Intestinal α1,2-fucosylatedProteins to Maintain Gut Homeostasis**

Ye Yao^1,2,^ ‡, Zhangming Pei^1,2,^ ‡, Yuanyuan Dai^3^, Yinghan Chen^1,2^, Zepeng Chang^4^, Hongchao Wang^1,2^, Jianxin Zhao^1,2,5^, Qixiao Zhai^1,2^, Wei Chen^1,2,6^, Wenwei Lu^1,2,5,6,*^

^1^ State Key Laboratory of Food Science and Resources, Jiangnan University, Wuxi

214122, China;

^2^ School of Food Science and Technology, Jiangnan University, Wuxi 214122, China;

^3^ Department of Gastroenterology, Affiliated Hospital of Jiangnan University, Wuxi 214122, China;

^4^ School of Biotechnology, Jiangnan University, Wuxi 214122, China;

^5^ MOE Medical Basic Research Innovation Center for Gut Microbiota and Chronic Diseases, School of Medicine, Jiangnan University, Wuxi 214122, China;

^6^ National Engineering Research Center for Functional Food, Jiangnan University,

Wuxi 214122, China;

^*^Correspondence: luwenwei@jiangnan.edu.cn

‡These authors have contributed equally to this work.


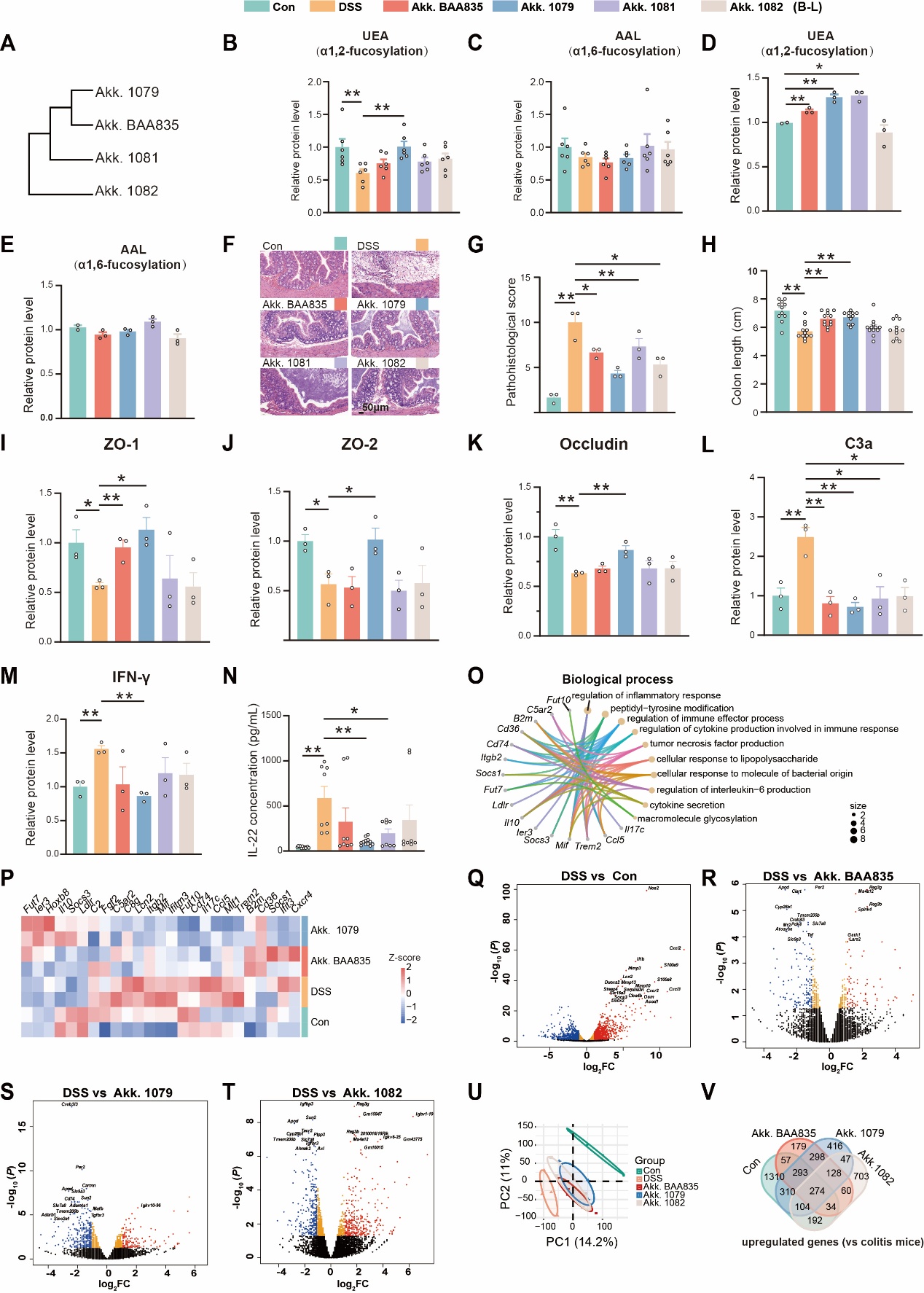


**Figure S1.** *Akkermansia muciniphila* affected colonic fucosylation, pathology, expression of barrier- and inflammation-related proteins, and modulated the transcription profiles of colons in colitis mice, related to Figure 1. A) Phylogenetic analysis based on the core-genome of four *A. muciniphila* strains. B-C) Relative colonic α1, 2-fucosylation (B) and α1, 6-fucosylation (C) levels in the colonic tissues of control mice, colitic mice, and those gavage-fed with *A. muciniphila* strains, *n* = 4. (D-E) Relative colonic α1, 2-fucosylation (D) and α1, 6-fucosylation (E) levels in HcoEpiC cells co-cultured with *A. muciniphila*, *n* = 2-3. F-H) Representative H&E colon sections (F), H&E pathohistological scores (G) and changes in colon lengths (H) of colons, *n* = 12. I-M) Relative protein levels of ZO-1 (I), ZO-2 (J), Occludin (K), C3a (L), IFN-γ (M) of colon tissues, *n* = 3. N) IL-22 concentrations in colons detected by ELISA, *n* = 8. O-P) Chord diagram presenting the representative biological processes (O) and heatmap showing the representative differentially expressed genes (P) of RAW264.7 cells treated with serum from control mice, colitis mice, and those administered by *A. muciniphila* BAA-835 and strain CCFM1079, *n* = 2*.* Q-T) Volcano plots illustrating the distinct gene expression patterns of the colons of colitis mice compared to those of control mice (Q), colitis mice administered with *A. muciniphila* strains BAA835 (R), CCFM1079 (S), and CCFM1082 (T) based on RNA-seq analysis. U) Principal component analysis based on the transcription levels of genes. V) Venn diagram analysis of up-regulated genes in control, *A. muciniphila*-treated colitis mice versus colitis mice. Error bar represents mean ± SEM. Statistical significance was determined by one-way analysis of variance (ANOVA) followed by Fisher’s LSD tests with adjustments for multiple comparisons (B-E, G-N). *, *P* < 0.05; **, *P* < 0.01.


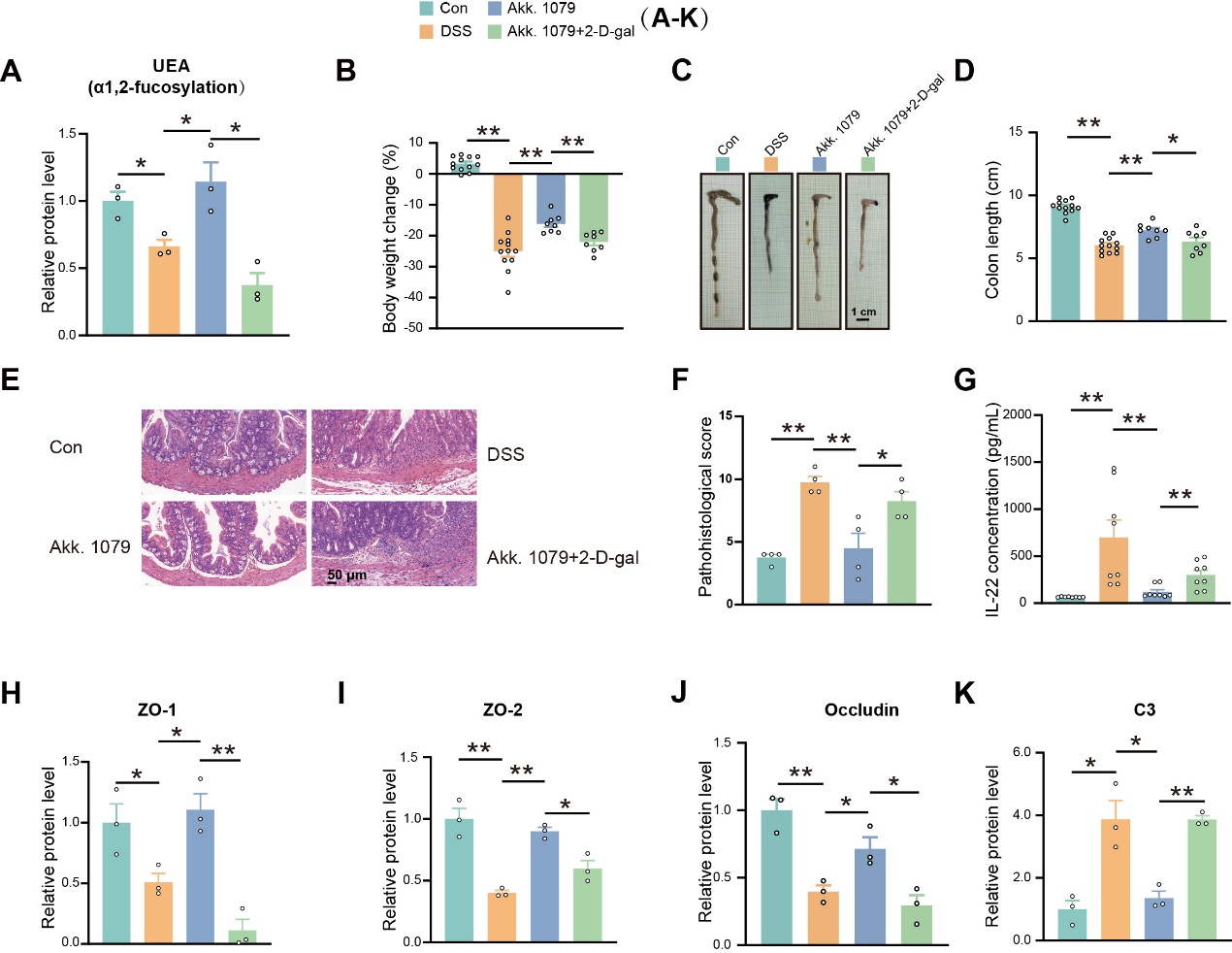


**Figure S2.** Inhibition of α1,2-fucosylation diminished the colitis-alleviating effects of *Akkermansia muciniphila* CCFM1079, related to Figure 1. A-G) Changes in α1, 2-fucosylation levels (A, *n* = 3), body weight (B, *n* = 8), colon length (C-D, *n* = 8), representative H&E sections of colons and pathohistological score (E-F, *n* = 4) of colitis mice gavaged with *A. muciniphila* CCFM1079 and injected by 2-deoxy-D-galactose. G) IL-22 concentrations in colons of mice detected by ELISA, *n* = 8. H-K) Relative protein levels of ZO-1 (H), ZO-2 (I), Occludin (J), C3 (K). Error bar represents mean ± SEM. Statistical significance was determined by one-way analysis of variance (ANOVA) followed by Fisher’s LSD tests with adjustments for multiple comparisons (A-B, D, F-K). *, *P* < 0.05; **, *P* < 0.01.


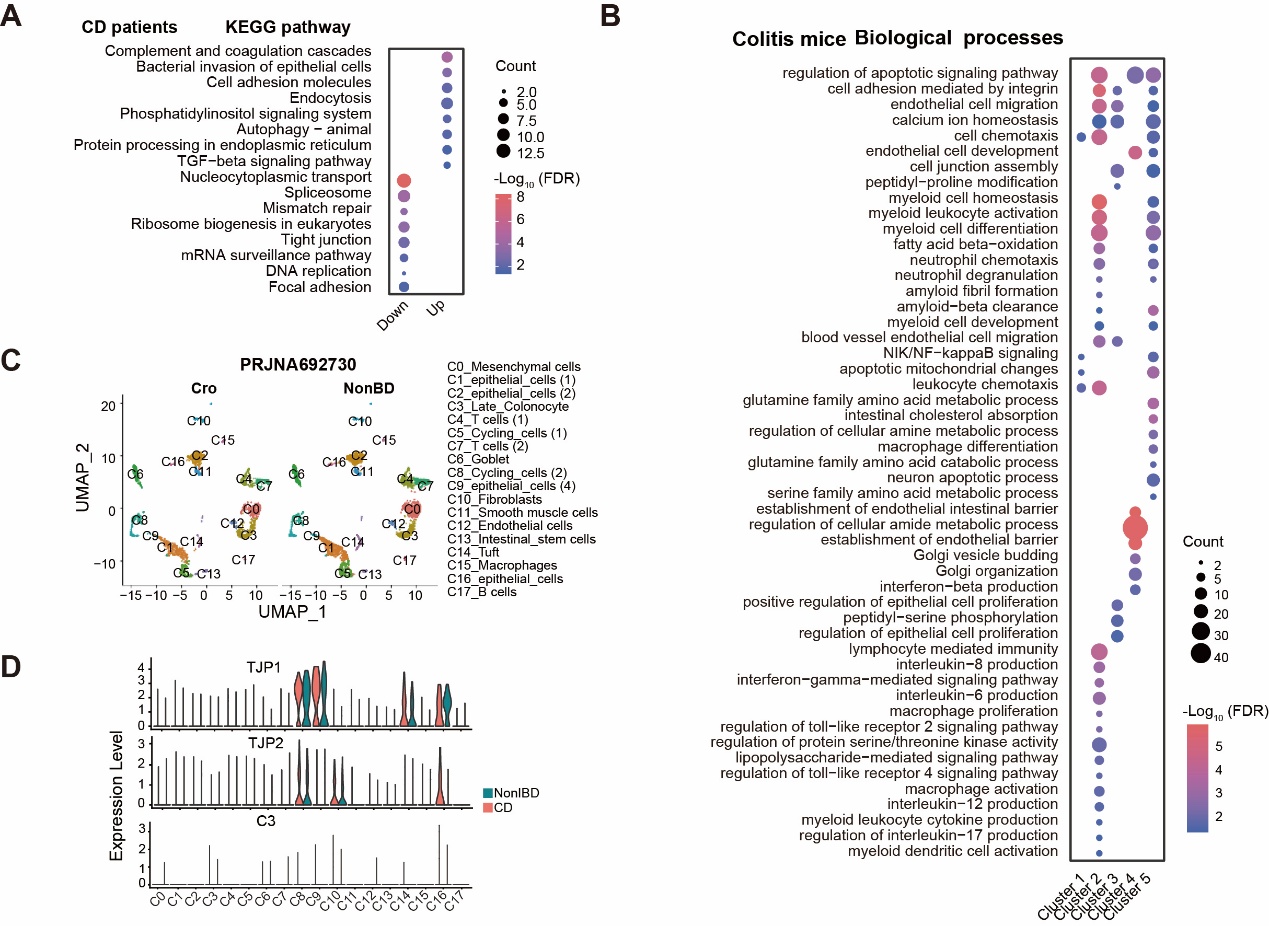


**Figure S3.** *Akkermansia muciniphila* modulates the α1,2-fucosylation of colonic proteins, related to Figure 2. A) Bubble plot showing representative KEGG pathways of differential expressed α1, 2-fucosylated proteins in colons of CD patients, *n* = 4. B) Bubble plot showing representative biological processes of genes encoding proteins in clusters 1-5. C) Uniform Manifold Approximation and Projection (UMAP) of cell clusters following cell type annotation using known cell type markers and highly enriched genes based on single-cell sequencing data of mucosal tissue from the ascending colons of treatment-naïve adult individuals with CD (*n* = 3) and non-IBD healthy controls (NonIBD, *n* = 4) (data from NCBI, PRJNA692730). D) Expression of *TJP1*, *TJP2*, *F11R* in 18 cell clusters.


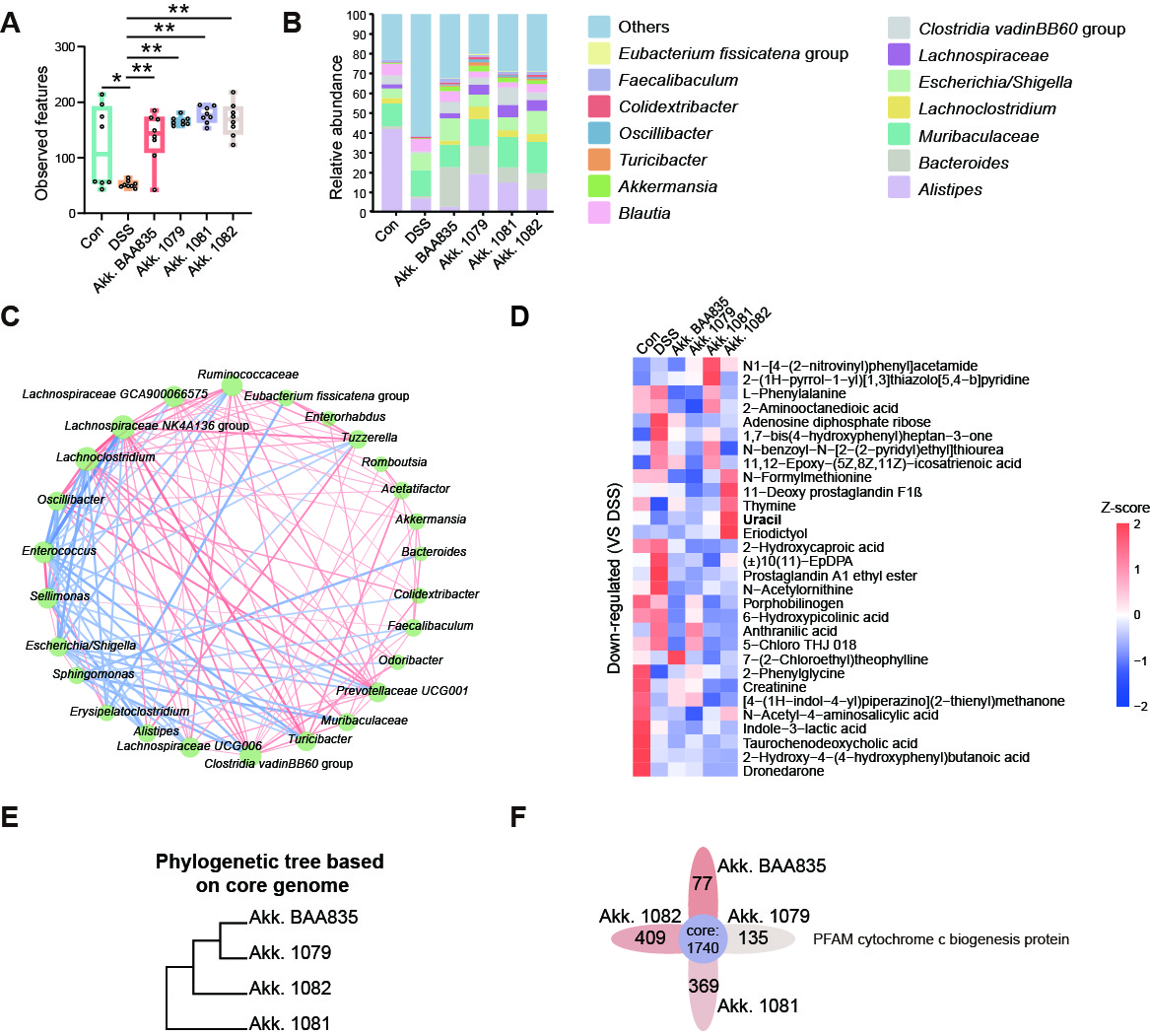


**Figure S4.** *Akkermansia muciniphila* changes the composition and metabolism of gut microbiota in cecal contents of colitis mice, related to Figure 3. A) Observed features of cecal microbiota of colitis mice gavaged with *A. muciniphila*, *n* = 8-12. B) Stacked bar plots showing the genus levels of cecal microbiota of colitis mice gavaged with *A. muciniphila*, *n* = 8-12. C) Network diagram showing the correlation of the cecal bacteria of colitis mice and those gavaged with *A. muciniphila*. The blue line represents a negative correlation while the red line represents a positive correlation. *n* = 8-12. D) Heatmap showing the relative concentration of downregulated cecal metabolites in *A. muciniphila-*treated colitis mice compared to untreated colitis mice. E) Phylogenetic tree based on the pan-genome of four *A. muciniphila* strains. F) The number of unique genes in four *A. muciniphila* strains. Non-normally distributed data is presented as Median (Interquartile Range, IQR) (A). Statistical significance was determined by Kruskal-Wallis tests followed by uncorrected Dunn's test with adjustments for multiple comparisons (A). *, *P* < 0.05; **, *P* < 0.01.


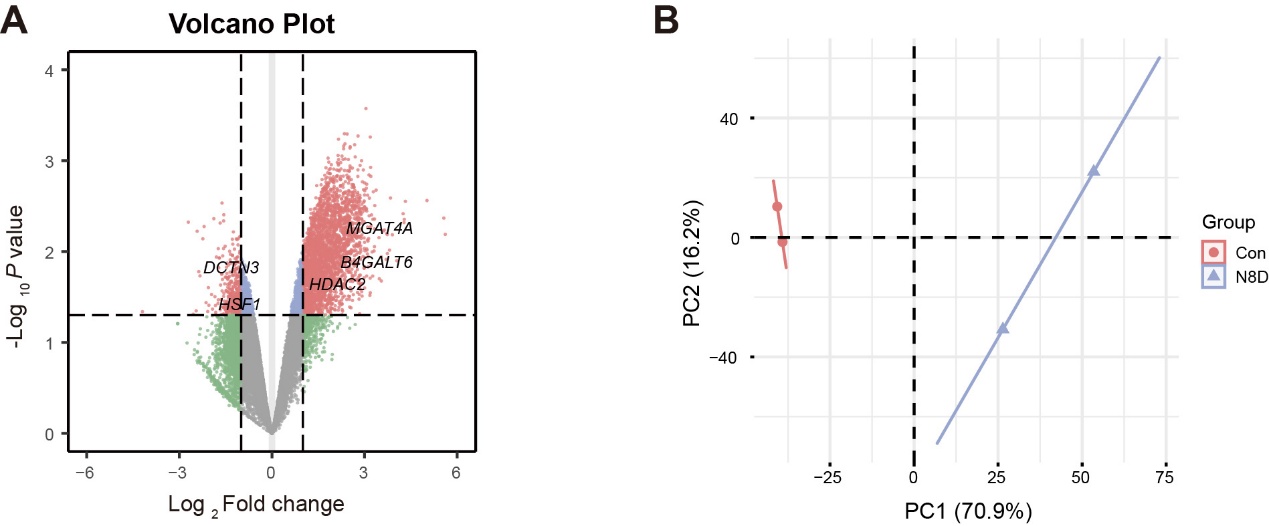


**Figure S5.** N8-acetyspermidine affects the gene expression of THP-1 cells, related to Figure 4. A) Volcano plot illustrating the distinct gene expression patterns of THP-1 cells treated with N8-acetyspermidine. B) Principal component analysis based on the transcription levels of genes of THP-1 cells treated with N8-acetyspermidine.


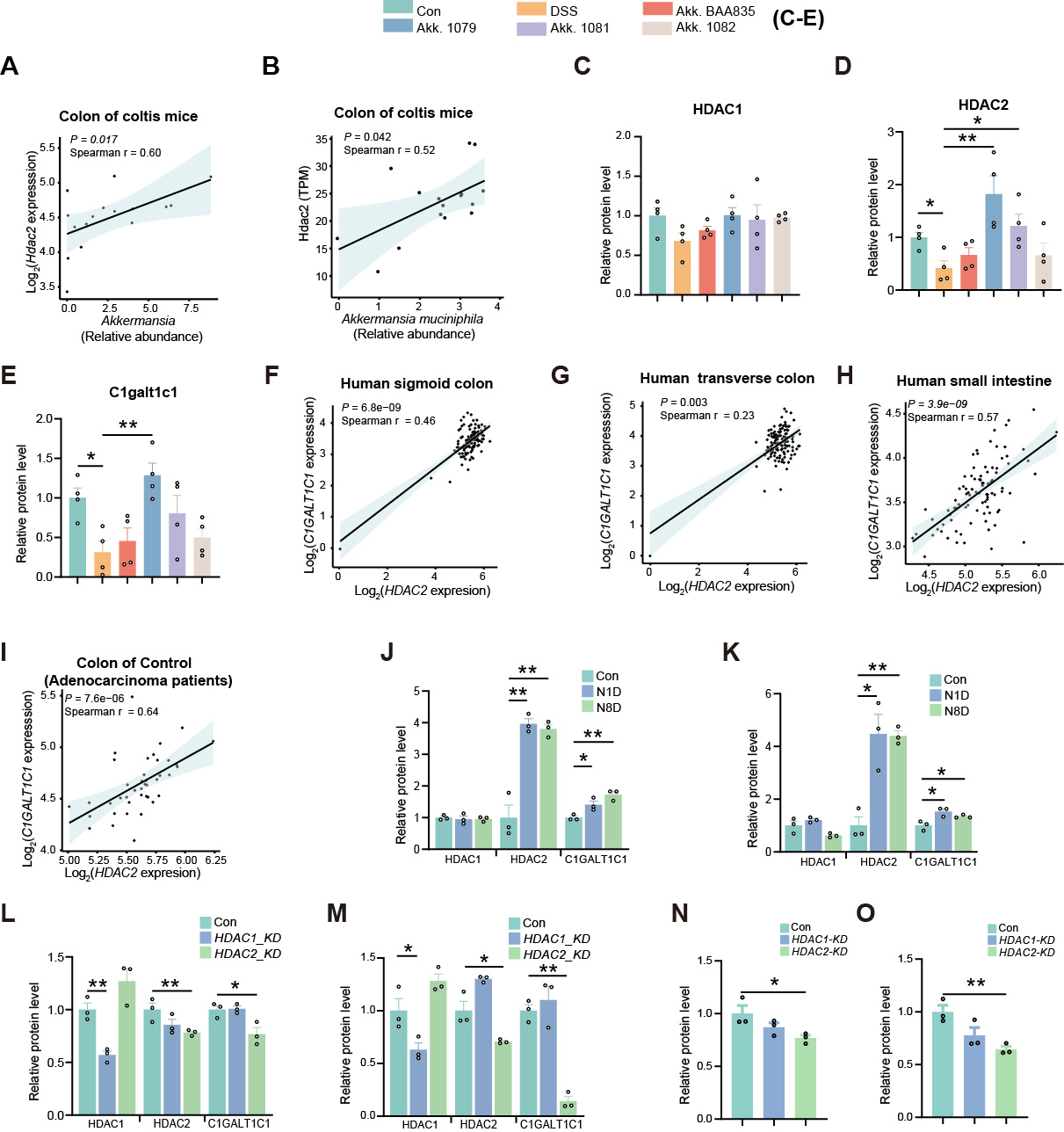


**Figure S6.** N-acetyspermidine upregulated the transcription of *C1GALT1C1* by elevating the *HDAC2* transcription, related to Figure 5. A) Dot plot depicting the correlation between the gene expression of *Hdac2* and the relative abundance of *Akkermansia* in the intestines of colitis mice and those administered with *A. muciniphila*. B) Dot plot showing the correlation between the gene transcription of *Hdac2* and the relative abundance of *A. muciniphila* in the intestines of colitis mice. C-E) Relative protein levels of HDAC1 (G), HDAC2 (H), C1GALT1C1 (I) in colonic tissues of colitis mice administered with *A. muciniphila* strains, *n* = 3-4. F-I) Dot plots showing the correlation between the gene transcription of *C1GALT1C1* and *HDAC2* in sigmoid colon (F), transverse colon (G), small intestine (H), and control of colon adenocarcinoma (I) based on data from the GTEx and TCGA normal database. J-K) Relative protein levels of HDAC1, HDAC2, C1GALT1C1 in HcoEpiC cells (J) and THP-1 cells (K) treated by N1-acetyspermidine (N1D) and N8-acetyspermidine (N8D), *n* = 3. L-M) Relative protein levels of HDAC1, HDAC2, C1GALT1C1 in HcoEpiC cells (L) and THP-1 cells (M) treated with SiRNA targeted to *HDAC1* and *HDAC2*, *n* = 3. N-O) Relative α1,2-fucosylation levels of HcoEpiC cells (N) and THP-1 cells (O) when *HDAC1* or *HDAC2* was knocked down, *n* = 3. Error bar represents mean ± SEM. Statistical significance was determined by one-way analysis of variance (ANOVA) followed by Fisher’s LSD tests with adjustments for multiple comparisons (G-J, K-M) and unpaired Student’s t test (K). *, *P* < 0.05; **, *P* < 0.01.


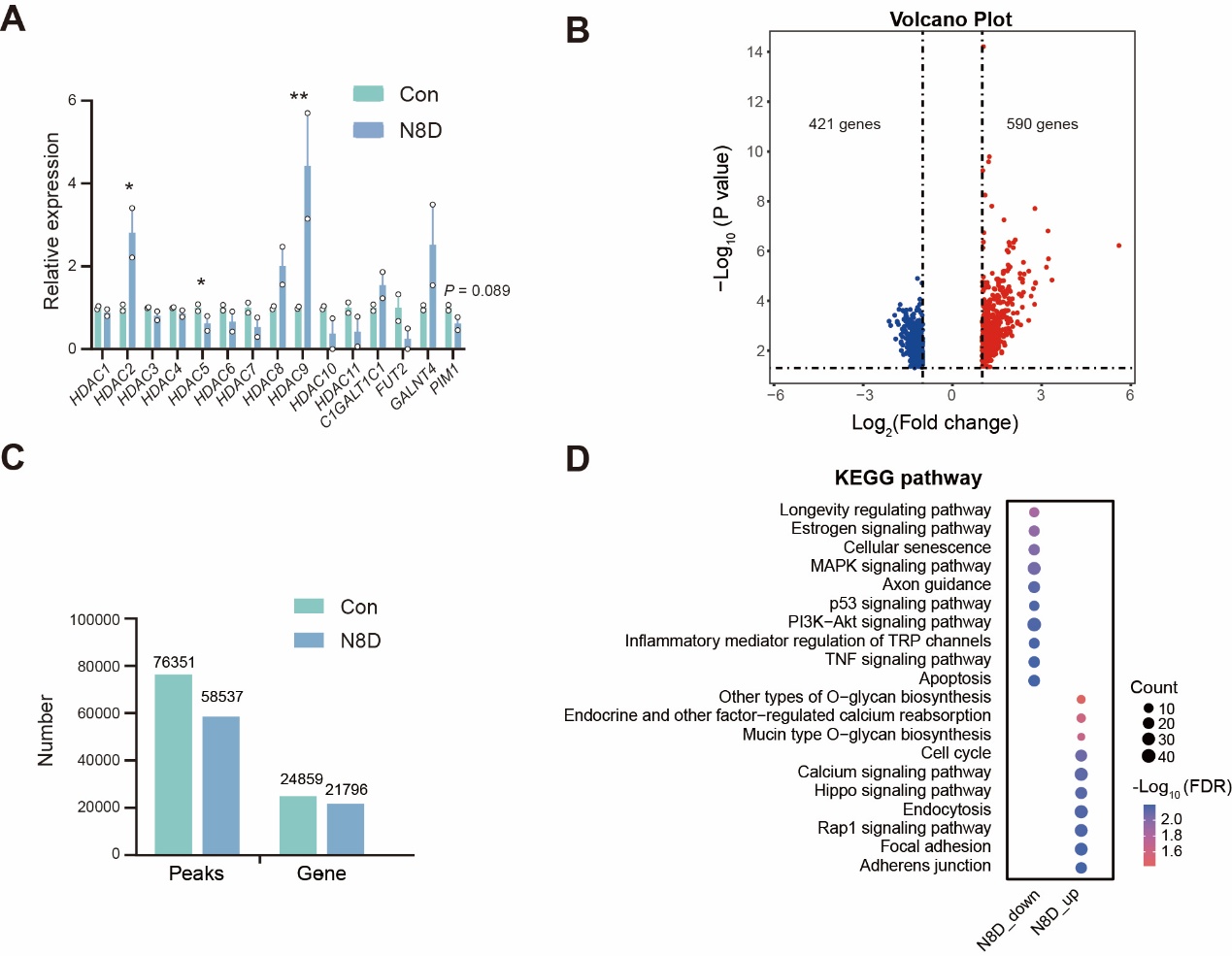


**Figure S7.** N-acetyspermidine affects the transcriptome and chromatin accessibility of THP-1 cells, related to Figure 6. A) The expression of genes of HDAC family and related to α1,2-fucosylation in THP-1 cells treated by N8D, *n* = 2. B) Volcano plots illustrating the patterns of peaks in control and N8D-treated THP-1 cells based on ATAC-sequencing analysis, *n* = 3. C) The number of peaks and genes of ATAC-seq in control and N8D-treated THP-1 cells. D) Bubble chart displaying the representative KEGG pathways of upregulated and downregulated genes in N8D-treated THP-1 cells.


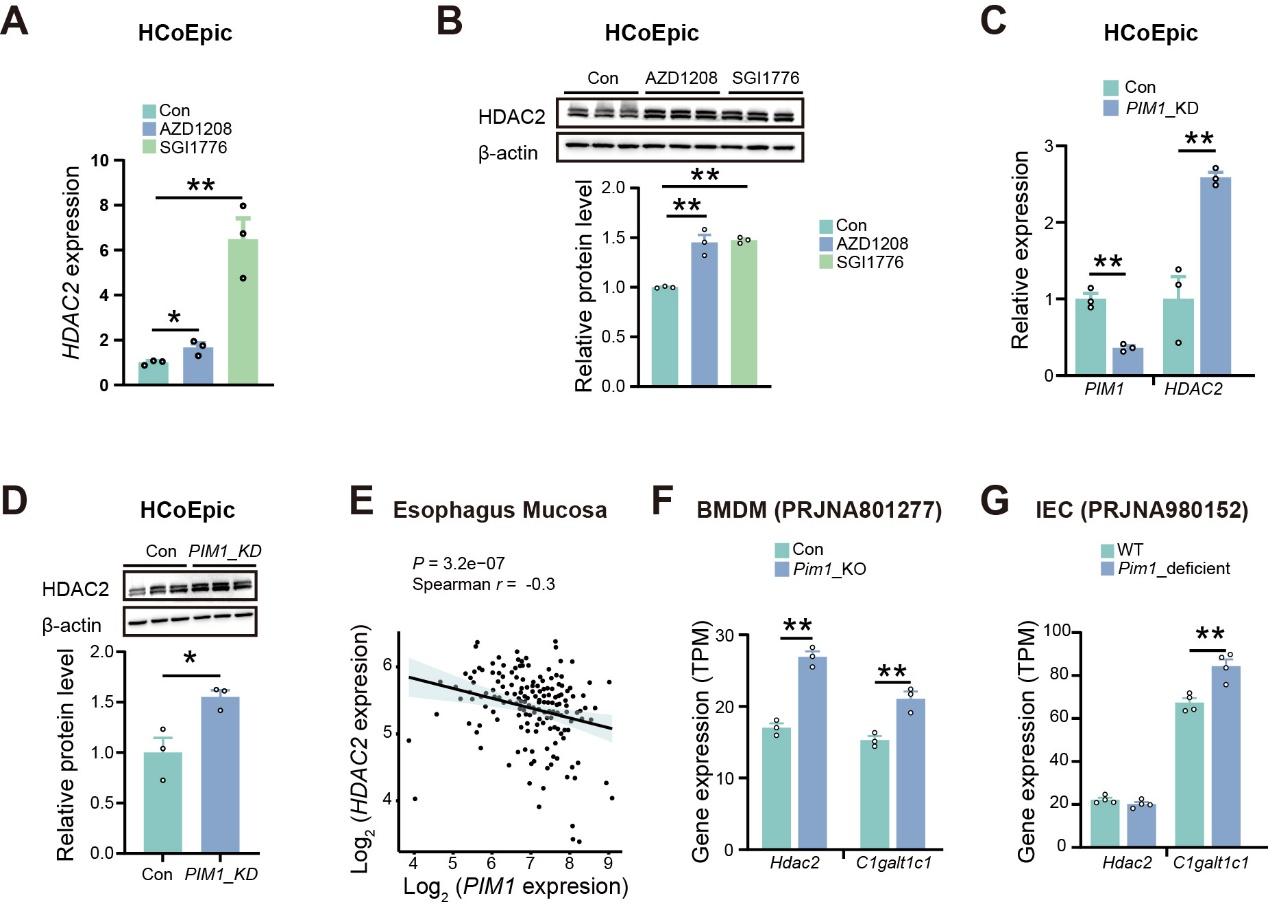


**Figure S8.** Inhibition of *PIM1* upregulated *HDAC2* expression, related to Figure 6**.** A) qPCR analysis of *HDAC2* in HCoEpic cells treated with PIM1 inhibitors AZD1208 and SGI1776, n=3. B) Immunoblot analysis of *HDAC2* in HCoEpic cells treated with PIM1 inhibitors AZD1208 and SGI1776, n=3. C) qPCR analysis of PIM1 and *HDAC2* in HCoEpic cells treated with siRNA targeted to PIM1, n=3. D) Immunoblot analysis of *HDAC2* in HCoEpic cells treated with siRNA targeted to PIM1, n=3. E) Dot plot depicting the correlation between the gene expression of *HDAC2* and *PIM1* in human esophagus mucosa, based on data from the GTEx database. F) Gene expression of *Hadc2* and *C1galt1c1* in WT and *Pim1*^−/−^ bone marrow-derived macrophages (BMDMs) (data from NCBI, PRJNA801277). G) Gene expression of *Hdac2* and *C1galt1c1* of intestinal epithelial cells (IEC) from WT mice and Pim1-deficient mice (data from NCBI, PRJNA980152). Error bar represents mean ± SEM. Statistical significance was determined by one-way analysis of variance (ANOVA) followed by Fisher’s LSD tests with adjustments for multiple comparisons (A-B) and unpaired Student’s t test (C-D, F-G). *, *P* < 0.05; **, *P* < 0.01.


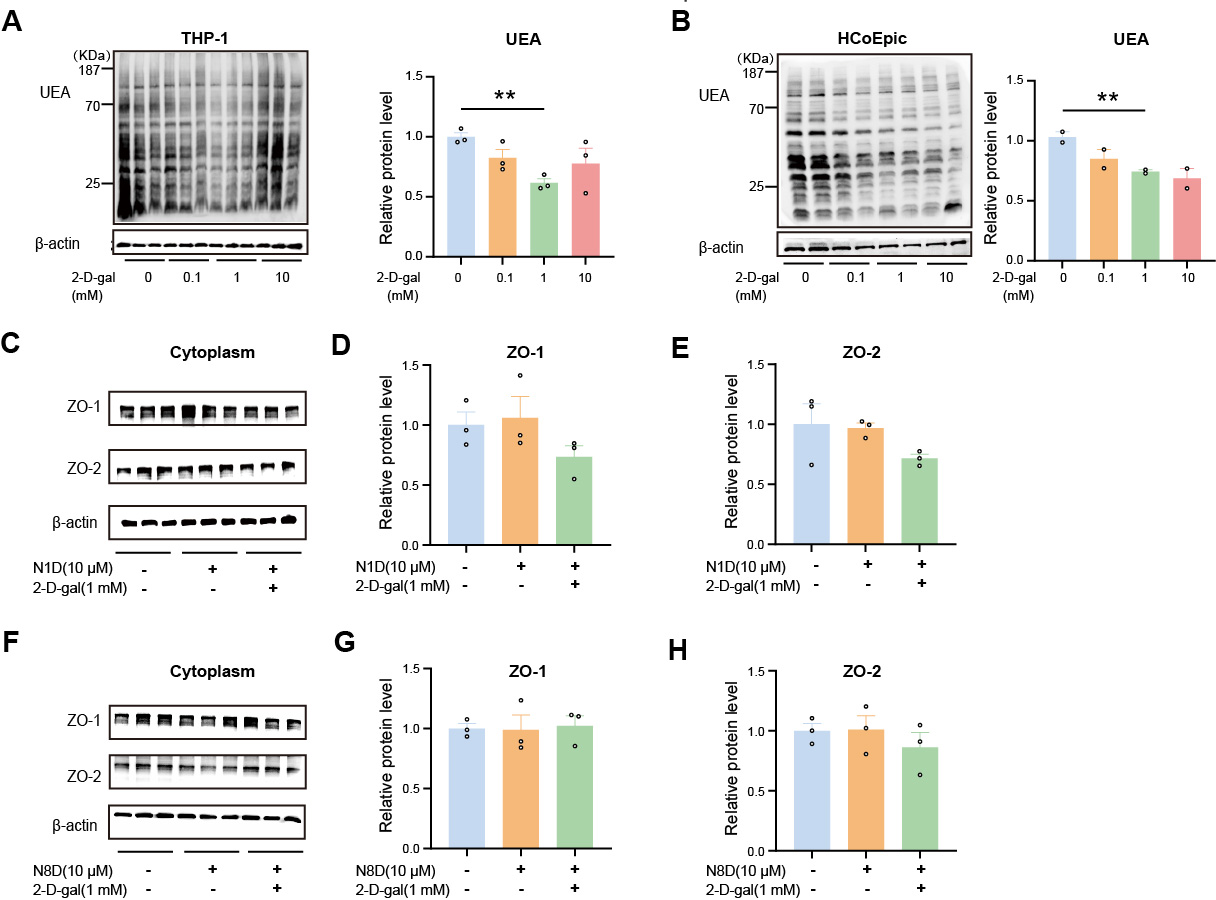


**Figure S9.** Inhibition of α1,2-fucosylation did not alter the levels of ZO-1 and ZO-2 in the cytoplasm of HcoEpiC cells, related to Figure 7. A-B) Relative α1,2-fucosylation levels in HcoEpiC cells (A) and THP-1 cells (B) treated with 0, 0.1, 1, 10 mM 2-deoxy-D-galactose (2-D-gal), *n* = 3. C-E) Relative protein levels of ZO-1 (D) and ZO-2 (E) in the cytoplasm of HcoEpiC cells treated with N1-acetylspermidine (N1D) and 1 mM 2-D-gal, *n* = 3. F-H) Relative protein levels of ZO-1 (G) and ZO-2 (H) in the cytoplasm of HcoEpiC cells treated with N8-acetylspermidine (N8D) and 1 mM 2-D-gal, *n* = 3. Error bar represents mean ± SEM. Statistical significance was determined by one-way analysis of variance (ANOVA) followed by Fisher’s LSD tests with adjustments for multiple comparisons (A-B, D-E, G-H). *, *P* < 0.05; **, *P* < 0.01.

**Table S1****.** The differentially expressed genes of control mice, colitis mice and those gavaged with *A. muciniphila* BAA835, strain CCFM1079, and strain CCFM1082.

**Table S2.** The differentially expressed α1,2-fucosylated proteins in colons of Crohn's disease patients and colitis mice administered with *A. muciniphila* BAA835, strain CCFM1079.

**Table S3.** The gene annotation of genes in the complete genomes of *Akkermansia muciniphila* CCFM179, strain CCFM1081, and strain CCFM1082

**Table S4.** The differentially expressed genes of THP-1cells treated by N8-acetylspermidine.

**Table S5.** The nucleosome-free regions (NFRs) with differential signals in THP-1cells treated by N8-acetylspermidine.

**Table S6.** Lowest free energy between HDAC2/PIM2 with ligands N1-acetyspermidine and N8-acetyspermidine

**Table S7.** The sequences of SiRNAs for the knockdown of *HDAC1* and *HDAC2*.

**Table S8.** The primers used in RT-qPCR.

**Table S9.** Key reagent and data accession numbers
