# Supplementary material for: Akkermansia muciniphila‐Derived N‐Acetylspermidine Modulates the Localization of Intestinal α1,2‐Fucosylated Proteins to Maintain Gut Homeostasis
Source: Adv Sci (Weinh). 2025 Aug 7;12(38):e06576. doi: 10.1002/advs.202506576 (PMC12520552; doi:10.1002/advs.202506576)
Supplement: Supplementary file 7 — Supplemental Table 6‐8 [file ADVS-12-e06576-s003.docx]

**Table S6** **Lowest free energy between HDAC2/PIM2 with ligands N1-acetyspermidine and N8-acetyspermidine.**

| Proteins | ligands | Lowest free energy (kcal/mol) |
| --- | --- | --- |
| HADC2 | N1-acetyspermidine | -4.06 |
|  | N8-acetyspermidine | -4.36 |
| PIM1 | N1-acetyspermidine | -7.82 |
|  | N8-acetyspermidine | -7.84 |

**Table S7 Sequences of siRNAs for the knockdown of *HDAC1* and *HDAC2***

| Target | SiRNA | Sequence (5′ to 3′) |
| --- | --- | --- |
| *HDAC1* | Sence | GGAUGUUGGAAAUUACUAUTT |
|  | Anti-sence | AUAGUAAUUUCCAACAUCCTT |
| *HDAC2* | Sence | GCAGAUGCAGAGAUUUAAUTT |
|  | Anti-sence | AUUAAAUCUCUGCAUCUGCTT |
| *PIM1* | Sence  Anti-sence | GCCCUGAGACCAUCAGAUATT  UAUCUGAUGGUCUCAGGGCTT |

**Table S8 Primers used in RT-qPCR**

| Target | Primers | Sequence (5′ to 3′) |
| --- | --- | --- |
| *HDAC1* | Forward | GGTCCAAATGCAGGCGATTCCT |
|  | Reverse | TCGGAGAACTCTTCCTCACAGG |
| *HDAC2* | Forward | CTCATGCACCTGGTGTCCAGAT |
|  | Reverse | GCTATCCGCTTGTCTGATGCTC |
| *C1GALT1C1* | Forward | CAGTTTGCCTGAAATATGCTGGAG |
|  | Reverse | CAGCCTTCTACTACCTGGTTGG |
| *PIM1* | Forward  Reverse | TCTACTCAGGCATCCGCGTCTC  CTTCAGCAGGACCACTTCCATG |
| *TP73* | Forward  Reverse | CATGGAGACGAGGACACGTACT  TGCCGATAGGAGTCCACCAGTG |
| *SP3* | Forward  Reverse | TGTCCCAACTGTAAAGAAGGTGG  CTCCAGAATGCCAACGCAGATG |
| *ACTB* | Forward | CACCATTGGCAATGAGCGGTTC |
|  | Reverse | AGGTCTTTGCGGATGTCCACGT |
| 27F and 1492R | Forward | AGAGTTTGATCCTGGCCTCA |
|  | Reverse | GGTTACCTTGTTACGACTT |
| 341F and 806R | Forward | CCTAYGGGRBGCASCAG |
|  | Reverse | GGACTACNNGGGTATCTAAT |
| *Akkermansia muciniphila* | Forward | CAGCACGTGAAGGTGGGGAC |
|  | Reverse | CCTTGCGGTTGGCTTCAGAT |
| Universal bacteria | Forward | GTGSTGCAYGGYTGTCGTCA |
|  | Reverse | ACGTCRTCCMCACCTTCCTC |
