# Supplementary material for: Akkermansia muciniphila‐Derived N‐Acetylspermidine Modulates the Localization of Intestinal α1,2‐Fucosylated Proteins to Maintain Gut Homeostasis
Source: Adv Sci (Weinh). 2025 Aug 7;12(38):e06576. doi: 10.1002/advs.202506576 (PMC12520552; doi:10.1002/advs.202506576)
Supplement: Supplementary file 8 — Supplemental Table 9 [file ADVS-12-e06576-s004.docx]

**Table S9 Key reagent and data accession numbers**

| REAGENT or RESOURCE | SOURCE | IDENTIFIER |
| --- | --- | --- |
| Antibodies |  |  |
| Anti-ZO-1 antibody | Abcam | Cat#ab96587;  RRID: AB_10680012 |
| Anti-ZO-1 antibody (IP) | Abcam | Cat# ab276131;  RRID: AB_3083081 |
| Anti-ZO-2 antibody | Cell Signaling Technology | Cat#2847S;  RRID: AB_2203575 |
| Anti-C3 antibody | Abcam | Cat#ab200999;  RRID: AB_2924273 |
| Anti-C3 antibody (IP) | Abcam | Cat# ab97462;  RRID: AB_10679468 |
| Anti-Occludin antibody | Abcam | Cat#ab216327;  RRID: AB_2737295 |
| Anti-IFN-γ antibody | Thermo Fisher Scientific | Cat#MM700B;  RRID: AB_223608 |
| Anti-C1GALT1C1 antibody | Proteintech | Cat#19254-1-AP;  RRID: AB_10638003 |
| Anti-HDAC1 antibody | Abcam | Cat#ab280198 |
| Anti-HDAC2 antibody | Abcam | Cat#ab219053 |
| Anti-β-actin antibody | Cell Signaling Technology | Cat#3700S |
| Anti-GAPDH antibody | Cell Signaling Technology | Cat#5174T |
| Anti-Sodium Potassium ATPaseATPase antibody | Abcam | Cat#ab76020;  RRID: AB_1310695 |
| HRP-conjugated Affinipure Goat Anti-Mouse IgG(H+L) | Proteintech | Cat#SA00001-1;  RRID: AB_2722565 |
| HRP-conjugated Affinipure Goat Anti-Rabbit IgG(H+L) | Proteintech | Cat#SA00001-2;  RRID: AB_2722564 |
| HRP-conjugated Affinipure Goat Anti-Rat IgG(H+L) | Proteintech | Cat#SA00001-15;  RRID: AB_2864369 |
| biotinylated Aleuria Aurantia Lectin | Vector laboratories | Cat#B-1395-1 |
| Agarose bound Ulex Europaeus Agglutinin I | Vector laboratories | Cat#AL-1063-2 |
| Rabbit IgG Isotype Control | Absin | Cat# abs172294 |
| Bacterial strains |  |  |
| *Akkermansia muciniphila* BAA-835 | American Type Culture Collection | N/A |
| *Akkermansia muciniphila* CCFM1079 | Culture Collection of Food Microorganisms (CCFM), Jiangnan University | N/A |
| *Akkermansia muciniphila* CCFM1081 | CCFM, Jiangnan University | N/A |
| *Akkermansia muciniphila* CCFM1082 | CCFM, Jiangnan University | N/A |
| Continued |  |  |
| REAGENT or RESOURCE | SOURCE | IDENTIFIER |
| Chemicals |  |  |
| Dextran sodium sulfate | MP Biomedicals | Cat#0216011080 |
| 2-deoxy-D-galactose | Sigma | Cat#259580 |
| N1-acetylspermidine | MedChemExpress | Cat#HY-113056A |
| N8-acetylspermidine | MACKLIN | Cat#N912364 |
| Fetal Bovine Serum | Gibco | Cat# 10099 |
| DMEM | Gibco | Cat#C11965500B |
| RPMI 1640 Medium | Gibco | Cat#C11875500B |
| Trizol | Life Technology | Cat#15596018 |
| Phorbol 12-Myristate 13-Acetate (PMA) | MedChemExpress | Cat#HY-18739 |
| Levamisole hydrochloride | Solarbio | Cat#L8230 |
| Ampicillin | Sangon Biotech | Cat#A100339 |
| Neomycin | Sangon Biotech | Cat#A610366 |
| Metronidazole | Sangon Biotech | Cat#A600633 |
| Vancomycin | Sangon Biotech | Cat#A600983-0001 |
| Protein A and proteins G agarose beads | Absin | Cat#abs955 |
| Critical commercial assays |  |  |
| ChamQ Universal SYBR qPCR Master Mix | Vazyme | Cat#Q711 |
| HiScript ® III All-in-one RT SuperMix Perfect for qPCR | Vazyme | Cat#R333 |
| lL-22 Quantikine ELlSA Kit | R&D systems | Cat#M2200 |
| Human C3a ELISA Kit | Beyotime | Cat#PC091 |
| Mem-PER™ Plus Membrane Protein Extraction Kit | Thermo Scientific | Cat#89842 |
| Fast DNA Spin Kit for Feces | MP Biomedicals | Cat#116570200 |
| RNAprep Pure Cell Kit | TIANGEN | Cat#DP419 |
| Amicon® Ultra Centrifugal Filter, 30 kDa MWCO | Millipore | Cat# UFC503008 |
| Deposited data |  | Accession numbers |
| Cecal 16S rRNA data of colitis mice gavaged with *A. muciniphila* | This paper | PRJCA026893 |
| Cecal 16S rRNA data of colitis mice gavaged with *A. muciniphila* CCFM1079 and intraperitoneally injected with 2-deoxy-D-galactose | This paper | PRJCA031861 |
| RNA-Seq data of THP-1 cells | This paper | PRJCA026921 |
| RNA-Seq data of RAW264.7 cells | This paper | PRJCA026182 |
| RNA-Seq data of mice colons | This paper | PRJCA026197 |
| ATAC-Seq data of THP-1 cells | This paper | PRJCA032250 |
| Complete gnomes of *A. muciniphila* strains | This paper | PRJCA026211 |
| Continued |  |  |
| REAGENT or RESOURCE | SOURCE | IDENTIFIER |
| RNA-seq data of intestinal epithelial cells in IBD patients | Park et al.^[1]^ | PRJEB24645 |
| RNA-seq data of rectal biopsies from pediatric IBD patients | Vanhove et al. ^[2]^ | PRJNA483949 |
| Intestinal macrophage RNA-seq of patients with colonic IBD | Dharmasiri et al.^[3]^ | PRJNA507632 |
| single-cell RNA-seq data of human inflamed intestine | Garrido-Trigo et al.^[4]^ | PRJNA985602 |
| RNA-Seq data of perianal fistula tissues | Giulia et al.^[5]^ | PRJNA659792 |
| RNA-Seq data of recal mucosal biopsies | Haberman et al.^[6]^ | PRJNA429769 |
| RNA-Seq data of HDAC1/2/3 knockout Hela cells | Li et al.^[7]^ | PRJNA979181 |
| RNA-Seq data of WT BMDMs and *Pim1*^-/-^ BMDMs | Ko et al.^[8]^ | PRJNA801277 |
| RNA-Seq data of IECs from WT and *Pim1*_deficient mice |  | PRJNA980152 |
| RNA-Seq data of TP73 knockout human TL-Om1 cells | Ong et al.^[9]^ | PRJNA751062 |
| ATAC data of *HDAC2-* knockdown BE2C cells | Zhang et al.^[10]^ | PRJNA837079 |
| Metagenomic data of feces from IBD patients | Hall et al.^[11]^ | PRJNA385949 |
| Metagenomic data of feces from *Fut2*^-/-^ mice | Zhou et al.^[12]^ | PRJNA614498 |
| Untargeted metabolomics of feces of patients with UC | Schirmer et al.^[13]^ | PR001596 |
| RNA-Seq data of LPS-treated THP-1 cells | Ansari et al.^[14]^ | PRJNA858047 |
| RNA-Seq data of LPS- stimulated THP-1 cells | Aznaourova et al.^[15]^ | PRJNA394134 |
| RNA-Seq data of PMA- stimulated THP-1 cells | Vollmers et al.^[16]^ | PRJNA632845 |
| RNA-Seq data of LPS- stimulated BMDMs | Yu et al.^[17]^ | PRJNA520989 |
| RNA-Seq data of β-glucan treated BMDMs | Stothers et al.^[18]^ | PRJNA728581 |
| RNA-Seq data of LPS-treated BMDMs | Yu et al.^[19]^ | PRJNA609623 |
| RNA-Seq data of LPS-stimulated BMDMs |  | PRJNA385311 |
| Single-cell sequencing data of IBD patients and control | Kanke et al.^[20]^ | PRJNA692730 |
| Experimental models: Cell lines |  |  |
| Mouse: C57BL/6J | Beijing Vital River Laboratory Animal Technology | N/A |
| *Caenorhabditis elegans* | SHANGHAI MODEL ORAGANISMS | N/A |
| RAW264.7 cells | ATCC | N/A |
| THP-1 cells | ATCC | N/A |
| HCoEpic cells | ATCC | N/A |
| Oligonucleotides |  |  |
| Primers for qPCR: see Table S1 | This paper | N/A |
| The sequences of SiRNAs: see Table S2 | This paper | N/A |
| Software and algorithms |  |  |
| GraphPad Prim10.1.2 | GraphPad Software | https://www.graphpad.com/ |
| Continued |  |  |
| REAGENT or RESOURCE | SOURCE | IDENTIFIER |
| QIIME 2 | Bolyen et al.^[21]^ | https://docs.qiime2.org/2020.8/ |
| LEfSe | Segata et al.^[22]^ | N/A |
| R | R Core Team | https://www.r-project.org/ |
| FeatureCounts | Liao et al.^[23]^ | N/A |
| STAR 2.7.0e | Dobin et al.^[24]^ | N/A |
| EdgeR 3.7 | McCarthy et al.^[25]^ | N/A |
| KneadData | N/A | https://github.com/biobakery/kneaddata |
| MetaPhlAn 4.1.0.73 | Blanco-Míguez et al.^[26]^ | N/A |
| HUMAnN 3.9 | Beghini et al.^[27]^ | N/A |
| FastQC 0.11.9 | Babraham Bioinformatics | https://www.bioinformatics.babraham.ac.uk/projects/fastqc/ |
| Prokka | Seemann^[28]^ | N/A |
| BPGA 1.3 | Chaudhari^[29]^ | N/A |

**References**

[1] I. Park, J. Jung, S. Lee, K. Park, J. W. Ryu, M. Y. Son, H. S. Cho and D. S. Kim. Characterization of terminal-ileal and colonic Crohn's disease in treatment-naive paediatric patients based on transcriptomic profile using logistic regression.Journal of Translational Medicine. 2021, 19,

[2] W. Vanhove, P. M. Peeters, D. Staelens, A. Schraenen, J. Van der Goten, I. Cleynen, S. De Schepper, L. Van Lommel, N. L. Reynaert, F. Schuit *et al*. Strong Upregulation of AIM2 and IFI16 Inflammasomes in the Mucosa of Patients with Active Inflammatory Bowel Disease.Inflammatory Bowel Diseases. 2015, 21, 2673-2682.

[3] S. Dharmasiri, E. M. Garrido-Martin, R. J. Harris, A. C. Bateman, J. E. Collins, J. R. F. Cummings and T. Sanchez-Elsner. Human intestinal macrophages are involved in the pathology of both ulcerative colitis and crohn disease.Inflammatory Bowel Diseases. 2021, 27, 1641-1652.

[4] A. Garrido-Trigo, A. M. Corraliza, M. Veny, I. Dotti, E. Melon-Ardanaz, A. Rill, H. L. Crowell, A. Corbi, V. Gudino, M. Esteller *et al*. Macrophage and neutrophil heterogeneity at single-cell spatial resolution in human inflammatory bowel disease.Nat Commun. 2023, 14,

[5] G. Rizzo, F. Rubbino, S. Elangovan, G. Sammarco, S. Lovisa, S. Restelli, S. E. P. Chavez, L. Massimino, L. Lamparelli, M. Paulis *et al*. Dysfunctional Extracellular Matrix Remodeling Supports Perianal Fistulizing Crohn's Disease by a Mechanoregulated Activation of the Epithelial-to-Mesenchymal Transition.Cellular and Molecular Gastroenterology and Hepatology. 2023, 15, 741-764.

[6] Y. Haberman, R. Karns, P. J. Dexheimer, M. Schirmer, J. Somekh, I. Jurickova, T. Braun, E. Novak, L. Bauman, M. H. Collins *et al*. Ulcerative colitis mucosal transcriptomes reveal mitochondriopathy and personalized mechanisms underlying disease severity and treatment response.Nat Commun. 2019, 10,

[7] J. L. Li, L. Lu, L. L. Liu, X. L. Ren, J. W. Chen, X. Z. Yin, Y. H. Xiao, J. W. Li, G. Wei, H. Huang *et al*. HDAC1/2/3 are major histone desuccinylases critical for promoter desuccinylation.Cell Discovery. 2023, 9,

[8] R. Ko, J. Seo, H. Park, N. Lee and S. Y. Lee. Pim1 promotes IFN-β production by interacting with IRF3.Experimental and Molecular Medicine. 2022, 54, 2092-2103.

[9] J. Z. Ong, R. Yokomori, R. W. J. Wong, T. K. Tan, R. Ueda, T. Ishida, S. Iida and T. Sanda. Requirement for *TP73* and genetic alterations originating from its intragenic super-enhancer in adult T-cell leukemia.Leukemia. 2022, 36, 2293-2305.

[10] Y. X. Zhang, D. Remillard, U. Onubogu, B. Karakyriakou, J. N. Asiaban, A. R. Ramos, K. Bowland, T. R. Bishop, P. A. Barta, S. Nance *et al*. Collateral lethality between *HDAC1* and *HDAC2* exploits cancer-specific NuRD complex vulnerabilities.Nature Structural & Molecular Biology. 2023, 30,

[11] A. B. Hall, M. Yassour, J. Sauk, A. Garner, X. F. Jiang, T. Arthur, G. K. Lagoudas, T. Vatanen, N. Fornelos, R. Wilson *et al*. A novel *Ruminococcus gnavus* clade enriched in inflammatory bowel disease patients.Genome Medicine. 2017, 9,

[12] R. Zhou, C. Llorente, J. Cao, L. S. Zaramela, S. Zeng, B. Gao, S. Z. Li, R. D. Welch, F. Q. Huang, L. W. Qi *et al*. Intestinal alpha1-2-fucosylation contributes to obesity and steatohepatitis in mice.Cell Mol Gastroenterol Hepatol. 2021, 12, 293-320.

[13] M. Schirmer, M. Strazar, J. Avila-Pacheco, D. F. Rojas-Tapias, E. M. Brown, E. Temple, A. Deik, K. Bullock, S. Jeanfavre, K. Pierce *et al*. Linking microbial genes to plasma and stool metabolites uncovers host-microbial interactions underlying ulcerative colitis disease course.Cell Host Microbe. 2024, 32,

[14] S. A. Ansari, W. Dantoft, J. Ruiz-Orera, A. P. Syed, S. Blachut, S. van Heesch, N. Hübner and N. H. Uhlenhaut. Integrative analysis of macrophage ribo-Seq and RNA-Seq data define glucocorticoid receptor regulated inflammatory response genes into distinct regulatory classes.Computational and Structural Biotechnology Journal. 2022, 20, 5622-5638.

[15] M. Aznaourova, H. Janga, S. Sefried, A. Kaufmann, J. Dorna, S. M. Volkers, P. Georg, M. Lechner, J. Hoppe, S. Dökel *et al*. Noncoding RNA *MaIL1* is an integral component of the TLR4-TRIF pathway.Proc Natl Acad Sci U S A. 2020, 117, 9042-9053.

[16] A. C. Vollmers, S. Covarrubias, D. Kuang, A. Shulkin, J. Iwuagwu, S. Katzman, R. Song, K. Viswanathan, C. Vollmers, E. Wakeland *et al*. A conserved long noncoding RNA, GAPLINC, modulates the immune response during endotoxic shock.Proc Natl Acad Sci U S A. 2021, 118,

[17] W. W. Yu, Z. Wang, K. L. Zhang, Z. X. Chi, T. Xu, D. L. Jiang, S. Chen, W. X. Li, X. Y. Yang, X. Zhang *et al*. One-Carbon Metabolism Supports S-Adenosylmethionine and Histone Methylation to Drive Inflammatory Macrophages.Molecular Cell. 2019, 75, 1147-+.

[18] C. L. Stothers, K. R. Burelbach, A. M. Owen, N. K. Patil, M. A. McBride, J. K. Bohannon, L. M. Luan, A. Hernandez, T. K. Patil, D. L. Williams *et al*. β-Glucan Induces Distinct and Protective Innate Immune Memory in Differentiated Macrophages.J Immunol. 2021, 207, 2785-2798.

[19] W. W. Yu, Z. Wang, X. F. Yu, Y. H. Zhao, Z. L. Xie, K. L. Zhang, Z. X. Chi, S. Chen, T. Xu, D. L. Jiang *et al*. Kir2.1-mediated membrane potential promotes nutrient acquisition and inflammation through regulation of nutrient transporters.Nat Commun. 2022, 13,

[20] M. Kanke, M. M. K. Ng, S. Connelly, M. Singh, M. Schaner, M. T. Shanahan, E. A. Wolber, C. Beasley, G. C. Lian, A. Jain *et al*. Single-Cell Analysis Reveals Unexpected Cellular Changes and Transposon Expression Signatures in the Colonic Epithelium of Treatment-Naive Adult Crohn's Disease Patients.Cellular and Molecular Gastroenterology and Hepatology. 2022, 13, 1717-1740.

[21] E. Bolyen, J. R. Rideout, M. R. Dillon, N. Bokulich, C. C. Abnet, G. A. Al-Ghalith, H. Alexander, E. J. Alm, M. Arumugam, F. Asnicar *et al*. Reproducible, interactive, scalable and extensible microbiome data science using QIIME 2.Nature Biotechnology. 2019, 37, 852-857.

[22] N. Segata, J. Izard, L. Waldron, D. Gevers, L. Miropolsky, W. S. Garrett and C. Huttenhower. Metagenomic biomarker discovery and explanation.Genome Biology. 2011, 12,

[23] Y. Liao, G. K. Smyth and W. Shi. featureCounts: an efficient general purpose program for assigning sequence reads to genomic features.Bioinformatics. 2014, 30, 923-930.

[24] A. Dobin, C. A. Davis, F. Schlesinger, J. Drenkow, C. Zaleski, S. Jha, P. Batut, M. Chaisson and T. R. Gingeras. STAR: ultrafast universal RNA-seq aligner.Bioinformatics. 2013, 29, 15-21.

[25] D. J. McCarthy, Y. S. Chen and G. K. Smyth. Differential expression analysis of multifactor RNA-Seq experiments with respect to biological variation.Nucleic Acids Res. 2012, 40, 4288-4297.

[26] A. Blanco-Míguez, F. Beghini, F. Cumbo, L. J. McIver, K. N. Thompson, M. Zolfo, P. Manghi, L. Dubois, K. D. Huang, A. M. Thomas *et al*. Extending and improving metagenomic taxonomic profiling with uncharacterized species using MetaPhlAn 4.Nature Biotechnology. 2023, 41, 1633-1644.

[27] F. Beghini, L. J. McIver, A. Blanco-Míguez, L. Dubois, F. Asnicar, S. Maharjan, A. Mailyan, P. Manghi, M. Scholz, A. M. Thomas *et al*. Integrating taxonomic, functional, and strain-level profiling of diverse microbial communities with bioBakery 3.Elife. 2021, 10,

[28] T. Seemann. Prokka: rapid prokaryotic genome annotation.Bioinformatics. 2014, 30, 2068-2069.

[29] N. M. Chaudhari, V. K. Gupta and C. Dutta. BPGA- an ultra-fast pan-genome analysis pipeline.Sci Rep. 2016, 6,
